# Computational Screening of Metal–Organic Framework Membranes for the Separation of 15 Gas Mixtures

**DOI:** 10.3390/nano9030467

**Published:** 2019-03-20

**Authors:** Wenyuan Yang, Hong Liang, Feng Peng, Zili Liu, Jie Liu, Zhiwei Qiao

**Affiliations:** 1 Guangzhou Key Laboratory for New Energy and Green Catalysis, School of Chemistry and Chemical Engineering, Guangzhou University, Guangzhou 510006, China; 2111705055@e.gzhu.edu.cn (W.Y.); lhong@gzhu.edu.cn (H.L.); fpeng@gzhu.edu.cn (F.P.); gzdxlzl@gmail.com (Z.L.); 2 School of Chemistry and Chemical Engineering, South China University of Technology, Guangzhou 510640, China; 3 School of Chemistry and Chemical Engineering, Wuhan University of Technology, Wuhan 430072, China; ljie@wit.edu.cn

**Keywords:** metal–organic framework, gas separation, machine learning, molecular simulation, linear dimension reduction

## Abstract

The Monte Carlo and molecular dynamics simulations are employed to screen the separation performance of 6013 computation-ready, experimental metal–organic framework membranes (CoRE-MOFMs) for 15 binary gas mixtures. After the univariate analysis, principal component analysis is used to reduce 44 performance metrics of 15 mixtures to a 10-dimension set. Then, four machine learning algorithms (decision tree, random forest, support vector machine, and back propagation neural network) are combined with *k* times repeated *k*-fold cross-validation to predict and analyze the relationships between six structural feature descriptors and 10 principal components. Based on the linear correlation value *R* and the root mean square error predicted by the machine learning algorithm, the random forest algorithm is the most suitable for the prediction of the separation performance of CoRE-MOFMs. One descriptor, pore limiting diameter, possesses the highest weight importance for each principal component index. Finally, the 30 best CoRE-MOFMs for each binary gas mixture are screened out. The high-throughput computational screening and the microanalysis of high-dimensional performance metrics can provide guidance for experimental research through the relationships between the multi-structure variables and multi-performance variables.

## 1. Introduction

With the rapid development of the social economy, people increasingly depend on energy; however, energy is not inexhaustible. In recent years, the acceleration of the energy crisis has prompted people to think about how to use cleaner, more environmentally friendly, more efficient energy. Separation technology plays an indispensable part in the chemical industry, and is widely used in medicine, food, petroleum, chemical engineering, metallurgy, and other fields. However, separation also consumes energy, especially high-throughput gas separation; for example, deep cryogenic separation was used to separate N_2_/O_2_ in industry [1], where the energy consumption is much higher and the recovery rate is lower. In addition, chemical and physical absorption methods are used to remove acidic components (H_2_S and CO_2_) from CH_4_ in industry, including the low-temperature methanol, alcohol amine, and alkali methods. Energy consumption is extremely high during the regeneration of the absorbent [2,3]. The hydrate method was adopted to separate CO_2_ and N_2_ from flue gas and recovery from fuel gas in the industry [4,5], but the synthesis conditions are harsh, and the synthesis rate is slow. In the past 10 years, many researchers have proposed that membrane separation can greatly reduce the energy consumption [6,7] and operate simply, as well as be environmentally safe, low cost, less polluting, efficient, and scalable. However, it is not easy to find an excellent membrane material. In recent years, Yang et al. [8] greatly enhanced the diffusivity of CO_2_ by introducing amine functionalized polymer membranes (porous polymers diethylenediamine (PP-DETA) and porous polymers 1,2-dimethylethylenediamine (PP-menm)), thus increasing the permselectivity of CO_2_/CH_4_. Jusoh et al. [9] researched the separation performance of CO_2_/CH_4_ by T/6FDA-durene polyimide loaded with 1 wt% zeolite membrane. They found that the permeability of CO_2_ and selectivity of CO_2_/CH_4_ were 80% and 172% higher those attained using pristine 6FDA-durene. However, as reported, polymeric membranes may include several limitations, such as their inherent low permeability/selectivity, and inferior chemical and thermal stability [10,11]. Therefore, it is necessary to find a new high-performance membrane, which is proving to be a bottleneck in development.

Recently, a new type of material has attracted the attention of researchers: metal–organic frameworks (MOFs) that are self-assembled by organic links and inorganic metal ions [12]. MOFs are widely used in adsorption [13], separation [14], storage [15,16], and catalysis [17] because of their large specific surface area (500 to 6500 m^2^/g), wide pore size (1 to 98 Å), unsaturated metal sites, and structural properties [18]. Tens of thousands of MOFs have been synthesized to date, and most of them have been applied in gas separation. For example, Hou et al. [19] prepared ZIF-8/g-C_3_N_4_ membranes that were hybrids of a two-dimensional graphite carbonitride (g-C_3_N_4_) nanosheet and ZIF-8. Comparing with the original ZIF-8 membrane, the performance of the hybrid membrane was improved; the selectivity of H_2_/CO_2_ can reach 42. Wang et al. [20] researched nano-thick MOF membranes composed of porous two-dimensional metal–organic nanosheets, which shortened the gas transport pathway; thus, it exhibited H_2_ permeance that was three times higher than that of the graphene oxide (GO) membranes. The open and parallel pores in the nano-thick molecular sieving membranes improve the gas concentration gradient and further allow high gas permeation flux and high selectivity. However, conventional experiments consume a lot of time, energy, and cost. During the experiment, reagents and drugs can harm laboratory staff, and it is impossible to conduct experimental tests on thousands of MOFs. Therefore, in order to improve research efficiency, many researchers have proposed high-throughput computer simulations with artificial intelligence to screen and predict MOFs with excellent performance [21,22]. These algorithms address the influence of the geometry and energy feature descriptors on the separation performance of the MOF. Seda et al. have done a lot of research into calculations on metal–organic framework membranes (MOFMs). Avci et al. [23] screened the permselectivity of 3875 MOFMs for H_2_/CO_2_ with simulations. The results showed that the selectivity of H_2_/CO_2_ and the permeability of H_2_ were computed as 2.1 × 10^−5^ to 6.3 and 230 to 1.7 × 10^6^ Barrer. Daglar et al. [24] studied the separation effect of 3806 MOFMs for CO_2_/N_2_. The result showed that 15 optimal MOFMs with the selectivity of CO_2_/N_2_ is between 15–820, and the permeability of CO_2_ is 1.19 × 10^5^ to 1.95 × 10^6^ Barrer when the mixture gas of CO_2_/N_2_ (15/85) is at 1 bar and 298 K. The optimal MOFMs had a narrow geometric range (3.75 < pore limiting diameters (PLD) < 4.5 Å, *φ* < 0.75, volumetric surface area (VSA) < 1000 m^2^/g). The selectivity, diffusion, permeability of computation-ready, experimental metal–organic framework membranes (CoRE-MOFMs) for CO_2_/N_2_/CH_4_ were simulated in previous work [25], and seven optimal MOFMs were screened out. An optimal path was obtained with PLD (2.91–3.26 Å) and PSD_2.4 to 3.5 Å_ (48.2–64.1%). Sholl et al. [26] researched the properties of adsorption and diffusion of 1263 MOFMs for CO_2_/N_2_, and analyzed the effect between the geometry descriptors versus the properties of adsorption and diffusion.

Almost all of the reported studies outline only the separation of MOFMs for gases in energy fields, especially the separation of smaller diameter gases such as H_2_. However, many membrane materials are discouraged for other gas-mixing components that need to be separated in production. In this work, we conducted experiments on 15 two-component gas mixtures for separation requirements in production, and sought to find the best separation performance for these gases on CoRE-MOFMs. The 15 binary gas mixtures are as follows: CO_2_/CH_4_, CO_2_/H_2_S, CO_2_/N_2_, H_2_/CH_4_, H_2_/CO_2_, H_2_/N_2_, H_2_S/CH_4_, H_2_/O_2_, He/CH_4_, He/CO_2_, He/H_2_, He/N_2_, N_2_/CH_4_, O_2_/N_2_, and He/O_2_. Commercial gas membrane separations include O_2_/N_2_, CO_2_/CH_4_, H_2_/N_2_, He/air, and H_2_/CH_4_. CO_2_/CH_4_ and H_2_S/CH_4_ membrane separation is used to purify natural gas. The membrane separation of H_2_ is employed in hydrocracker purge, hydrotreater purge, and H_2_ recovery in refineries and ammonia plants [27,28]. Acid gas (CO_2_ and H_2_S) are needed to be removed from natural gas. The separation of N_2_ from air is also necessary, and N_2_ has been widely used as a protective gas [29]. The separation of He or pure He gas is applied in various fields, for example: silicon wafer manufacturing, the inerting of hydrogen fuel lines for rockets, arc welding, nuclear magnetic resonance machines, and accelerators [30].

The simulation process consisted of six parts. First, we simulated 6013 CoRE-MOFMs by the Monte Carlo (MC) method and molecular dynamics (MD), and calculated the six feature descriptors and 44 performance metrics. Second, we analyzed the relationship between the six feature descriptors and 44 performance metrics. Third, we reduced the dimensions of the 44 performance metrics by principal component analysis (PCA). Fourth, we predicted six feature descriptors and separation performance metrics, and analyzed them using four machine learning algorithms. Fifth, we analyzed the relative importance of the six feature descriptors. Sixth, we screened out the CoRE-MOFMs with better separation performance for mixed gas components.

## 2. Model and Methods

### 2.1. Model

In this work, the crystal structures of 6013 CoRE-MOFs were computationally screened; then, this database was refined after removing free solvent molecules and structural parameters that had been derived from the experimental data by Chung et al. [31,32]. We calculated the framework structures of the CoRE-MOFs after removing the solvent molecules. The atomic frameworks of the MOFs are described by the Lennard–Jones (LJ) and the electrostatic potentials:

(1) $$\sum 4\varepsilon_{ij}\left[{\left(\frac{\sigma_{ij}}{r_{ij}}\right)}^{12}-{\left(\frac{\sigma_{ij}}{r_{ij}}\right)}^{6}\right]+\sum \frac{q_{i}q_{j}}{4\pi \varepsilon_{0}r_{ij}}$$

where *ε_ij_* represents well depth; *σ_ij_* represents the equilibrium distances; *q_i_*, *q_j_* is the atomic charge of atoms *i* and *j*, respectively; and *ε*_0_ = 8.8542 × 10^−12^ C^2^N^−1^, representing the vacuum electric constant. The LJ potential parameters of all the MOFs come from the universal force field (UFF) [33], and are listed in Table S1. The atomic charge of MOFs is estimated quickly by the MOF electrostatic-potential-optimized charge scheme (MEPO-Qeq) method [34]. The UFF accurately predicted the adsorption and diffusion of MOFs, and was verified by the comparison with experimental data in previous work [24]. The structural descriptors of the MOFs were characterized by PLD (Å), large cavity diameter (LCD (Å)), VSA (m^2^/cm^3^), porosity *φ*, pore size distribution (PSD%_(2.5 to 3.5 Å)_) and MOF density (*ρ* (kg/m^3^)). We calculated the PLD and LCD in the Zeo++ package [35]. We calculated VSA using N_2_ with a diameter of 3.64 Å and the *φ* using He with a diameter of 2.58 Å as probes under the RASPA software package [36], respectively. We calculated the *Q*^0^_st_ of each gas by *NVT*-MC (*N* is the number of particles, *V* is the volume of system, *T* is the temperature of the system) with a single gas molecule in an infinite diluted state using 10^5^ moves of Widom insertion in the RASPA software package [36].

To construct seven adsorption gas components (CH_4_, N_2_, H_2_S, O_2_, CO_2_, H_2_, and He), we derived the force field parameters of the seven gas components listed in Table S2 from the transferable potentials for phase equilibria (TraPPE) force field [37]. CH_4_ is the joint atomic model. N_2_ was considered to be a three-point model with the 1.10 Å bond length for N–N. H_2_S was a four-bit model with a 1.13 Å bond length for S–H and LJ potential energy on the atoms of S and H. In addition, a virtual atom was located near the S atom, and the H atoms and the virtual atom were partially charged, whereas the S atom was not charged [38]. O_2_ was a three-point atom. For CO_2_, the bond length of C–O was 1.16 Å, and ∠OCO was 180°. The bond length of He was 2.58 Å.

### 2.2. Methods

The Henry’s constants *K* and diffusivities *D* of CH_4_, N_2_, H_2_S, O_2_, CO_2_, and H_2_ in 6013 CoRE-MOFMs were respectively estimated by MC and MD simulations with the same ensemble, NVT. The time step of MD simulation was 1 fs, and the temperature was controlled at 298 K by the Andersen thermostat. In principle, a single gas molecule should be added into a MOF to mimic infinite dilution. To improve the statistics, 30 gas molecules were used; however, gas–gas intermolecular interaction was switched off. The cross-interactions between MOFs and adsorbent molecules were calculated by Lorentz–Berthelot rules. In each simulation, the MOF structures were kept rigid. The periodic boundaries were applied in a three-dimensional system, and the cells were simulated and expanded to at least 24 Å in three-dimensional directions, respectively. A spherical cut-off of 12.0 Å with long-range correction was used to calculate the LJ interactions, whereas the electrostatic interactions were calculated using the Ewald sum. The electrostatic interaction between the frame molecule and the gas molecule was calculated by using Ewald summation. In each MOF, the MC simulation was run 10^5^ cycles with the first 50,000 cycles for equilibration and the last 50,000 cycles for ensemble average. The MD duration in each MOF was 2 ns, with the last 1 ns for production. All of MC and MD simulations were carried using the RASPA software package [36]. Through the adsorption and diffusion, the permeation was estimated.

## 3. Results

### 3.1. Map for Project Structure

**Figure 1:**
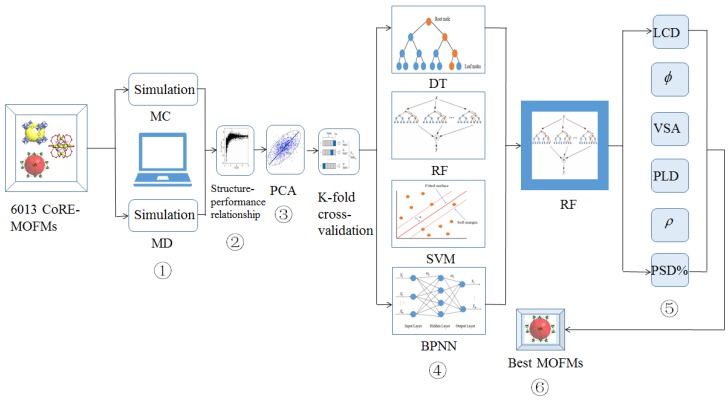
Schematic diagram of the project framework.

The research framework of this project is shown in Figure 1. We combined molecular simulation and various artificial intelligence algorithms to speed up the screening and accurate prediction of the gas separation performance of CoRE-MOFMs. We divided the research into six parts for the gas separation on MOFMs: (1) the separation performance of 15 different binary mixed gases were simulated by MC and MD on 6013 CoRE-MOFMs. For each CoRE-MOFM, we calculated six descriptors—LCD, *φ*, VSA, PLD, *ρ*, and PSD%_(2.5 to 3.5 Å)_—corresponding to permeability and permselectivity. (2) We analyzed the relationships between the geometrical descriptors and diffusion coefficients, diffusion selectivity, permeability, and permselectivity. (3) We analyzed the performance metrics and selected more than 85% of the top 10 principal components to cover all of the data variability information as a measure performance, including seven gas diffusion coefficients, 15 mixed gas diffusion selectivities, seven gas permeabilities, and 15 permselectivities using PCA dimensionality reduction. (4) To obtain a suitable artificial intelligence algorithm model for our materials and systems, we applied *k* times repeated *k*-fold cross-validation [21] to evaluate the predicted performance using four machine-learning methods: decision tree (DT), random forest (RF), support vector machine (SVM), and back propagation neural network (BPNN). We calculated the average linear correlation *R* and root mean square error (RMSE) as predictive criteria for machine learning. (5) By comparing and analyzing the four machine learning algorithms, we found that RF has the most accurate predicted effects and smaller errors. For further analysis, we calculated the relative importance of the six characteristic descriptors for the separation of 15 mixed component gases on CoRE-MOFMs. (6) Finally, based on the diffusion coefficient, diffusion selectivity, permeability, and permselectivity of 15 different gas components on CoRE-MOFMs, we selected the 30 optimal CoRE-MOFMs (the abbreviations are listed in SI).

### 3.2. Feature Descriptors and Performance Metrics

**Figure 2:**
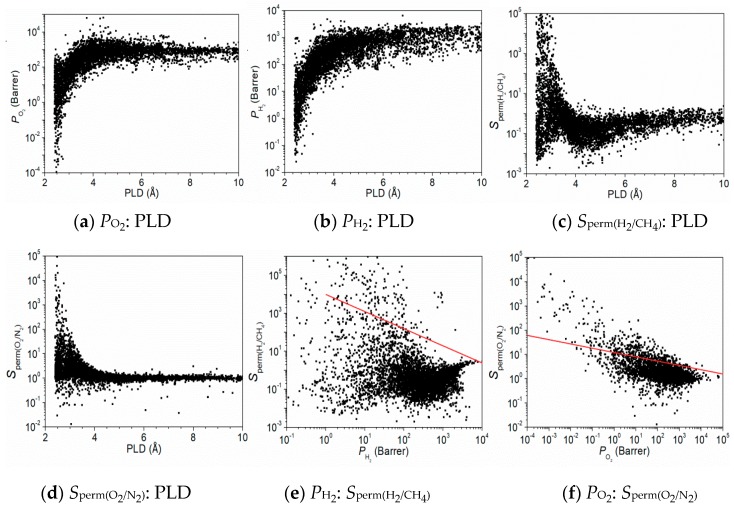
Relationships among (**a**) *P*_O_2__, (**b**) *P*_H_2__, and pore limiting diameter (PLD); relationships among (**c**) *S*_perm(H_2_/CH_4_)_, (**d**) *S*_perm(O_2_/N_2_)_, and PLD; relationship between (**e**) *S*_perm(H_2_/CH_4_)_ and *P*_H_2__ and (**f**) *S*_perm(O_2_/N_2_)_ and *P*_O_2__; the red line represents Robeson’s penetration data based on a wide range of polymer film upper bounds [39].

Figure 2a,b show the relationship between *P*_O_2__ (or *P*_H_2__) and PLD, where *P_i_* = *K_i_* × *D_i_*, *P_i_* represents the permeability, *K_i_* represents the Henry’s constant of the gas component, and *D_i_* is the diffusion coefficient. The amount of permeability of pure components was considered in this work. Comparing with a N_2_/CO_2_/CH_4_ mixture in our previous work [25], the *D_i_* and *P_i_* for each gas are increased, because the adsorbed amount and hence steric hindrance exist in real mixture. When the PLD is between 2.5–3.5 Å, *P*_O_2__ and *P*_H_2__ increase significantly with the increase of PLD. However, when PLD > 3.5 Å, *P*_O_2__ and *P*_H_2__ tend to be in equilibrium above 100 Barrer. The study showed that when PLD > 3.5 Å, both O_2_ and H_2_ could enter the MOFMs, because the pore sizes of O_2_ and H_2_ are <3.5 Å. The other five gases also exhibited similar trends, as shown in Figure S4. For *φ*, the relationship between permeability and *φ* had a similar trend to PLD, except that He and H_2_ showed a monotonic upward trend, and the slope ratio of H_2_ to *φ* was smaller than that of He to *φ*. Comparing with the *φ*, when *ρ* > 3000 kg/m^3^, the dispersions of *D* and *P* are very high, as shown in Figure S2d(1–7). The reason is that the MOFs indeed possess a relatively larger free space, but they consist of very heavy metal atoms (e.g. gold, platinum, uranium), and hence the *ρ* value appears to be high. Thus, it is hard for *ρ* to indicate the performance of CoRE-MOFMs. Compared with the influence of PLD on permeability, the diffusion coefficient showed a similar trend, but the value of the diffusion coefficient remained small. The diffusion coefficient rises sharply when PLD < 3.5 Å, and finally reaches stability when PLD > 6.0 Å, as shown in Figure S2. The main reason is strong steric hindrance when the gas diameter is close to the pore size of the MOFs. Such a relationship between PLD and *D* is similar to that in previous work [25]. Figure 2c,d exhibited a relationship between the permselectivity of two mixtures gas (H_2_/CH_4_ and O_2_/N_2_) and PLD, where *S*_perm(*i*/*j*)_ = *S*_adsp(*i*/*j*)_ × *S*_diff(*i*/*j*)_; and *S*_adsp(*i*/*j*)_ and *S*_diff(*i*/*j*)_ represent the adsorption selectivity and diffusion selectivity of components *i* and *j*, respectively. We found that the permselectivity of H_2_/CH_4_ had a maximum when PLD was between 2.5–3.5 Å, but a minimum value also existed within the same range. When PLD > 3.5 Å, the permselectivity of H_2_/CH_4_ first decreased and then increased, and finally tended toward equilibrium, as shown in Figure 2c. The results indicated that PLD was a key, but not perfect, separation performance index for H_2_/CH_4_. The permselectivity of O_2_/N_2_ decreased with increasing PLD, and finally approached equilibrium, as shown in Figure 2d. The results demonstrated that gas molecules reached a stable state exhibiting low permselectivity when PLD increased. The O_2_ molecules are neither adsorbed nor diffused by CoRE-MOFMs when the gas molecule is similar in size to the pore size of the CoRE-MOFMs. There was also a similar trend between the PLD and diffusion selectivity, as shown in Figure S3. Moreover, the *S*_diff_ are highly dispersed in the figure, because gas diffusion is influenced by many factors. Take for example, CO_2_/CH_4_: one the one hand, because CO_2_ has a stronger affinity than CH_4_ with any MOF, CO_2_ diffusion is retarded, which causes *S*_diff(CO_2_/CH_4_)_ < 1; on the other hand, CO_2_ has a smaller diameter than CH_4_, which causes *S*_diff(CO_2_/CH_4_)_ > 1. Upon analyzing the VSA and *ρ*, they showed no clear effect on the permselectivity of each gas component. By comparing the effect of *φ* and VSA with the diffusion selectivity, we observed roughly a straight line inclined toward the lower right, as shown in Figure S3. This is attributed to a small free space available in a MOF at a small *φ* or VSA for gas molecules to permeate, thus leading to hindering the gas molecule with a larger diameter. Additionally, since the VSA was determined using N_2_ as a probe with a diameter of 3.64 Å, thus, when the VSA of a MOF is zero or small, the smaller gas could pass the MOF such as CO_2_ (3.3 Å) and H_2_S (3.6 Å). Through a comprehensive analysis, Figure 2a–d reveal that the PLD had good separation performance for some gas components within 2.5–3.5 Å. It also was revealed that the gas components had a strong bond energy relationship with CoRE-MOFMs when entering the membrane material, which indicated adsorbed selectivity or diffusion selectivity. Therefore, the permselectivity was large when the pore size of the membrane material was small. Figure 2e,f illustrate the relationship between the permselectivity of H_2_/CH_4_ and O_2_/N_2_ versus *P*_H_2__ and *P*_O_2__. The red line in Figure 2e,f represented Robeson’s penetration data based on a wide range of polymer film upper bounds. The membrane materials above the red line were given priority. We found that the permselectivity and *P*_H_2__ were not monotonous. The permselectivity either increased slightly or decreased slightly, which was followed by a small increase as the permeability increased. The relationships among the permeability, permselectivity of other gas components, and PLD are shown in Figure S6.

### 3.3. Machine Learning

**Figure 3:**
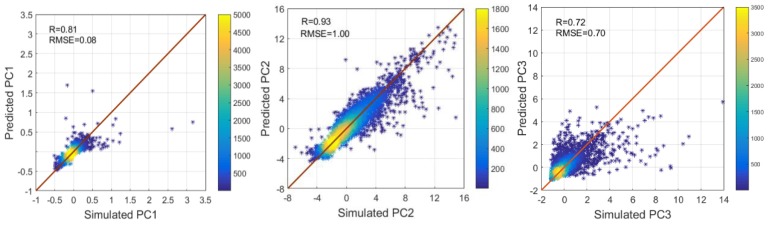
Predicted performance of the first three principal components (PC1, PC2, PC3) by the machine learning algorithm model of random forest (RF) versus the simulated results of computation-ready, experimental metal–organic framework membranes (CoRE-MOFMs) on the test set. The color of the point represents the amount of materials.

To screen structural variables with strong effects for all gas components and further predict the performance of new MOFMs, we weighed and analyzed 44 performance metrics using intelligent algorithms. In this work, we used PCA to reduce the dimensions of these 44 performance metrics, the details of PCA are in SI. We regarded the principal component as all of the largest indexes that covered the variation information by more than 85%. We selected the first 10 dimensions (87%) for further analysis. The percentage of variation information corresponding to each principal component is shown in Figure S7. In the study, six structural descriptors and 10 principal components were trained, tested, and analyzed after standardization. We randomly divided all of the data into *k* by using *k* times repeated *k*-fold cross-validation, where *k* = 5, in which one set was the test set, and the remaining four were training sets; see details in the Supplementary Information (SI). We repeated each training five times, and then each group of data was trained and predicted by four machine learning methods (DT, SVM, BPNN, and RF); see details in the SI. We calculated the average of *R* (the linear correlation coefficient) and the RMSE, which indicated the percentage of each principal component contributing to the data variation, from the training model. These were taken as the criterion for the algorithm prediction. The details of *R* and RMSE are shown in Table 1. The evaluation formula for the effect of the algorithm model is listed in Equations (2) and (3). Table S4 shows that the 10 principal components (*R =* 0.397) were larger and the RMSE = 0.619 was smaller in RF than in the other models. The DT algorithm model has better predictive performance with *R* = 0.575 and RMSE = 0.435. RF is composed of multiple DT, and is the improvement and optimization for DT. In the RF algorithm, a small change in the independent variable has no appreciable effect on the response variable. In addition, RF could make up for the weakness of the generalization of DT. However, it is easy for the RF model to cause over-fitting when the noise is big. Although the BPNN does not have better predictive performance for this system, it has a nonlinear mapping capability and self-study ability, which is suitable for complex internal mechanisms. The algorithm model is selected and the parameter is set accurately, which is very important for the prediction of different systems. The formulas are given in Equations (4) and (5). The results showed that the RF model had a better prediction performance, and was most suitable for the material system of this project. Figure 3 proved that the predicted performance for the first three principal components (PC1, PC2, and PC3) by the machine learning algorithm model of RF agreed well with the simulated results of CoRE-MOFMs. 

The prediction of the other three methods (DT, SVM, BPNN) is shown in Figure S7. The principal component did not have a better effect for predictions with larger variation information in Figure 3. *R* = 0.81 for the prediction of PC1, which was smaller than *R* = 0.93 for the prediction of PC2. However, the value of PC2 was generally larger than that of PC1 after standardization. Therefore, RMSE = 1 of PC2 was much larger than RMSE = 0.08 of PC1. The prediction performance of PC3 was lower than that of PC1 and PC2 (*R* = 0.72 and RMSE = 0.70 in PC3). We compared the three other machine learning algorithms (DT, SVM, and BPNN), and found that RF had the best prediction performance in the first three principal components, as well as synthesizing 10 principal components.

(2) $$R_{\mathrm{sum}}=0.293\cdot R_{\mathrm{PC}1}+0.187\cdot R_{\mathrm{PC}2}+\cdots +0.024\cdot R_{\mathrm{PC}10}$$

(3) $${\mathrm{RMSE}}_{\mathrm{sum}}=0.293\cdot {\mathrm{RMSE}}_{\mathrm{PC}1}+0.187\cdot {\mathrm{RMSE}}_{\mathrm{PC}2}+\cdots +0.024\cdot {\mathrm{RMSE}}_{\mathrm{PC}10}$$

(4) $$R=\frac{\sum \left(X-\bar{X}\right)}{\left(\sqrt{\frac{1}{N}\left(X_{i}-\bar{X}\right)2}\right)\cdot \left(\sqrt{\sum_{i=1}^{n}{\left(Y_{i}-\bar{Y}\right)}^{2}}\right)}$$

(5) $$\mathrm{RMSE}=\sqrt{\frac{1}{N}\sum_{i=1}^{n}{\left(y_{\mathrm{sim}}-y_{\mathrm{pre}}\right)}^{2}}$$

**Figure 4:**
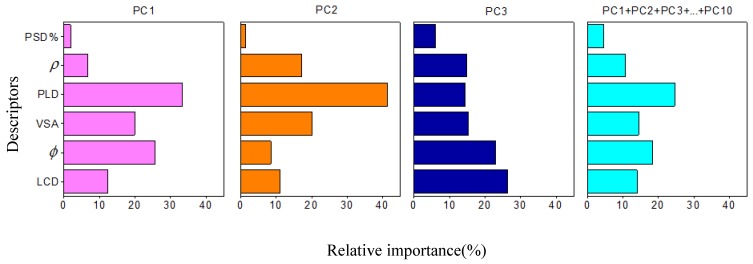
Relative importance of six feature descriptors versus PC1, PC2, PC3, PC1 + PC2 + ... + PC3 from the RF algorithm model.

These studies showed that machine learning had a good predictive effect on the principal components after dimension reduction. To further analyze the relative importance of the six feature descriptors versus the principal components, the RF was adopted. Based on the Gini coefficient, we calculated the relative importance of each feature descriptor. Figure 4 shows that the PLD had the largest weight for PC1 and PC2, which contained more variation information. The 10 principal component indexes showed that PLD had the highest weight. The relative importance of PLD was as follows: 0.33, 0.41, and 0.25. The results showed that PLD was the main factor affecting the separation performance of 15 mixed gases by CoRE-MOFMs; therefore, it should be considered first when researching the separation performance of these mixed components. When considering only the first three principal components (covering about 58% of the variation information), the relative importance of descriptors was as follows: PLD, *φ*, and *ρ*. When considering the top 10 principal components (covering about 87% of the variation information), the top four most important descriptors were as follows: PLD, *φ*, VSA, and LCD.

### 3.4. Best CoRE-MOFMs

To select the best CoRE-MOFMs, the benchmarks of permeability and permselectivity were set for each component of 15 binary gas mixtures, as listed in Table S5. Besides, to avoid the influence on the over-estimation of *K*, the benchmarks of diffusion selectivity were added, *S*_diff(CO_2_/CH_4_)_ > 10, *S*_diff(CO_2_/N_2_)_ > 10, *S*_diff(H_2_S/CH_4_)_ > 5, *S*_diff(CO_2_/H_2_S)_ > 10, as reported in the literature [26]. Based on these benchmarks, each binary gas mixture was preferably selected to the five best CoRE-MOFMs listed in Table S6, and the best two are listed in Table 2. Several repeated CoRE-MOFMs are noted in Table 2, indicating that these membranes have good separation properties for a variety of gas mixtures. Table 2 shows that the PLD was concentrated between 2.5–3.5 Å (80.0%). The results demonstrated that the gas molecules had stronger bond energy functions with the membrane materials. It was easier to distinguish among different sizes, and CoRE-MOFMs had better separation ability when the PLD was in a small range. Furthermore, a narrow range had a better separation effect when *φ* was between 0.1–0.3 (63.3%), indicating that *φ* also was a relatively important factor. Furthermore, there was a relatively comprehensive separation effect at smaller apertures when the LCD was between 3.3–5.0 Å (63.3%). These results were consistent with the final results of the intelligent algorithm model analyzed for the six feature descriptors. The PLD should be given priority, followed by *φ* and the LCD.

## 4. Conclusion

A high-throughput computational screening is used to calculate the separation performance of 6013 CoRE-MOFMs for 15 two-component gas mixtures; then, multiple intelligent algorithms are used to predict and analyze their structure­–property relationships. First, we used PCA to reduce 44 performance metrics to 10 dimensions, covering about 87% of all the variation information. The four machine learning algorithms (DT, RF, SVM, and BPNN) were optimized and evaluated using fivefold cross-validation, which each algorithm repeated five times. The results show that the RF algorithm better predicted the effect on the data of this project due to the smallest RMSE = 0.397 and largest *R* = 0.619. The *R* values for the first three principal components are 0.81, 0.93, and 0.72, and the RMSE values are 0.08, 1.00, and 0.70, respectively. Furthermore, we calculate the relative importance of six feature descriptors on the 10 performance metrics using the Gini coefficient by RF. The results show that the PLD is the most important feature descriptor. Analyzing the permeability and permselectivity of 15 gas components shows that a CoRE-MOFM with the PLD in a certain range (2.5 to 3.5 Å) has good permselectivity properties for mixed gas components, which also is suitable for a complex multicomponent gases mixture. In addition, *φ* and LCD also exhibit high relative importance on the separation of two-component gas mixtures. Finally, on the basis of the permeability and permselectivity, 30 optimal CoRE-MOFMs are identified as being suitable for the separation of different gas mixtures. This computational work by high-throughput screening and machine learning techniques gives the guideline for the development of MOF membranes for gas separation. 

## Figures and Tables

**Table 1:** The prediction of *R* values and root mean square error (RMSE) for principal component (PC1, PC2, PC3) on the algorithms model of four machine learning. DT: decision tree, SVM: support vector machine, BPNN: back propagation neural network.

ML	*R* Values			RMSE		
	PC1	PC2	PC3	PC1	PC2	PC3
**DT**	0.80	0.89	0.62	0.081	1.190	0.601
**RF**	0.81	0.93	0.72	0.080	1.000	0.700
**SVM**	0.79	0.89	0.63	0.080	1.210	0.790
**BPNN**	0.74	0.87	0.60	0.090	1.230	0.820

**Table 2:** Best CoRE-MOFMs. LCD: large cavity diameter, PLD: pore limiting diameter, PSD: pore size distribution, VSA: volumetric surface area, CSD: Cambridge structural database.

No.	*i*/*j*	CSD code	LCD (Å)	*φ*	VSA (m^2^/cm^3^)	PLD (Å)	*ρ* (g/m^3^)	PSD%	*P_i_* (Barrer)	*P_j_* (Barrer)	*S*_perm(*i*/*j*)_
1	CO_2_/CH_4_	XUZDUS *^c^*	4.25	0.16	15.50	2.99	1.82	3.31	1.26 × 10^9^	1.75 × 10^3^	7.16 × 10^5^
		XEJXER	4.12	0.19	4.33	3.58	1.60	0	1.33 × 10^8^	9.26 × 10^2^	1.44 × 10^5^
2	CO_2_/N_2_	ELUQIM06	2.89	0.04	0	2.41	1.80	100	7.20 × 10^6^	7.72 × 10^2^	9.32 × 10^3^
		NHBZZN10	3.41	0.08	0	2.94	1.54	68.84	1.57 × 10^4^	2.32 × 10^2^	67.63
3	H_2_/CO_2_	TUMGOX *^a^*	3.47	0.27	0	2.61	1.87	54.55	7.75 × 10^3^	1.76 × 10^3^	4.39
		HEDCEA	4.76	0.27	226.10	2.93	1.28	2.15	2.35 × 10^3^	5.95 × 10^2^	3.95
4	H_2_S/CH_4_	SEYFAE	4.14	0.28	87.08	3.31	2.03	0.43	5.57 × 10^4^	7.32 × 10^2^	76.12
		GUXPUL	2.79	0.02	0	2.58	1.60	100	4.11 × 10^4^	3.15 × 10^3^	13.07
5	H_2_/CH_4_	TESGUU	4.82	0.26	338.16	3.58	1.92	0	8.90 × 10^2^	0.07	1.20 × 10^4^
		ZIJVOF	5.29	0.43	622.86	3.32	1.23	0.10	9.35 × 10^2^	0.10	9.37 × 10^3^
6	H_2_/O_2_	TOWPAY *^b^*	3.47	0.27	0	2.61	1.87	54.55	7.75 × 10^3^	1.89 × 10^3^	4.10
		POWBIO	4.34	0.19	142.64	2.60	3.66	1.54	4.56 × 10^3^	1.18 × 10^3^	3.87
7	CO_2_/H_2_S	FAPYEA	2.53	0.00	0	2.46	1.58	100	1.93 × 10^9^	5.11 × 10^5^	3.78 × 10^3^
		XUZDUS *^c^*	4.25	0.16	15.50	2.99	1.82	3.31	1.26 × 10^9^	6.80 × 10^6^	1.85 × 10^2^
8	H_2_/N_2_	TUMGOX *^a^*	3.47	0.27	0	2.61	1.87	54.55	7.75 × 10^3^	2.01 × 10^3^	3.85
		DIMQOH	4.69	0.42	626.81	3.15	1.40	2.97	3.80 × 10^3^	1.12 × 10^3^	3.39
9	He/N_2_	TUMGOX *^a^*	3.47	0.27	0	2.61	1.87	54.55	7.11 × 10^3^	2.01 × 10^3^	3.53
		COWXOC *^d^*	5.83	0.30	517.90	2.91	1.28	0.57	7.79 × 10^3^	2.43 × 10^3^	3.21
10	He/H_2_	BUYNAL	4.62	0.47	617.88	3.97	0.92	0	20.34	5.12	3.97
		VULKOD	5.02	0.33	489.24	3.96	1.40	0.95	25.60	6.52	3.93
11	He/CH_4_	YEKWOC *^e^*	8.21	0.50	1099.57	3.05	1.28	0	1.21 × 10^2^	0.11	1.15 × 10^3^
		COXFOL	5.01	0.33	403.58	3.19	1.43	1.70	3.24 × 10^2^	7.48	43.32
12	N_2_/CH_4_	YEKWOC *^e^*	8.21	0.50	1099.57	3.05	1.28	0	6.82 × 10^3^	0.11	6.44 × 10^4^
		BAHGUN04	4.27	0.26	108.86	3.29	1.49	0	6.24 × 10^5^	11.11	5.62 × 10^4^
13	He/CO_2_	PUPNAQ	3.59	0.15	0	2.70	1.44	32.37	38.34	4.11	9.33
		TUMGOX *^a^*	3.47	0.27	0	2.61	1.87	54.55	7.11 × 10^3^	1.76 × 10^3^	4.03
14	O_2_/N_2_	GETXAG	3.33	0.10	0	2.61	1.54	99.99	1.11 × 10^4^	3.17 × 10^3^	3.48
		GOLQII	3.73	0.14	9.74	3.37	2.17	0	8.32 × 10^2^	2.48 × 10^2^	3.35
15	He/O_2_	COWXOC *^d^*	5.83	0.30	517.90	2.91	1.28	0.57	7.79 × 10^3^	2.07 × 10^3^	3.76
		TOWPAY *^b^*	3.47	0.27	0	2.61	1.87	54.55	7.11 × 10^3^	1.89 × 10^3^	3.76

*^a, b, c, d, e^* represent the same metal–organic framework membranes (MOFMs) for different binary gas mixtures.

## Supplementary Materials

The following are available online at https://www.mdpi.com/2079-4991/9/3/467/s1. Figure S1: Models of CH_4_, N_2_, H_2_S, O_2_, CO_2_, H_2_ and He. Figure S2: Relationships between diffusivity *D* and MOF descriptors. Figure S3: Relationships between diffusion selectivities *S*_diff_ and MOF descriptors. Figure S4: Relationships between permeability *P* and MOF descriptors. Figure S5: Relationships between permselectivity *S*_perm_ and MOF descriptors. Figure S6: Relationships between permeability *P* and permselectivity *S*_perm_. Figure S7: Predicted performance of the first three principal components (PC1, PC2, PC3) by three machine learning algorithm model of DT, SVM, and BPNN versus the simulated results of CoRE-MOFMs on the test set. Table S1: Lennard–Jones parameters of MOFs. Table S2: Lennard–Jones parameters and charges of adsorbates. Table S3: Principal component covering the ratio of variation information for 44 performance metrics. Table S4: RMSE and *R* versus four machine learning by synthesizing 10 principal components. Table S5: Benchmark of permeability and permselectivity for 15 gas mixtures. Table S6: Best CoRE-MOFMs for different gas mixtures. Abbreviation lists. Details of six mathematical analysis methods (*k* times repeated *k*-fold cross-validation, principal component analysis, decision tree, random forest, support vector machine, back propagation neural network).
