# Supplementary material for: Computational Screening of Metal–Organic Framework Membranes for the Separation of 15 Gas Mixtures
Source: Nanomaterials (Basel). 2019 Mar 20;9(3):467. doi: 10.3390/nano9030467 (PMC6474094; doi:10.3390/nano9030467)
Supplement: Supplementary file 1 [file nanomaterials-09-00467-s001.pdf]

Supplementary Materials

Computational Screening of Meta Organic Framework Membranes for the Separation of 15 Gas Mixtures

Wenyuan Yang [1], Hong Liang [1], Feng Peng [1,2], Zili Liu [1], Jie Liu [3] and Zhiwei Qiao [1,2,*]

1. Guangzhou Key Laboratory for New Energy and Green Catalysis, School of Chemistry and Chemical Engineering, Guangzhou University, Guangzhou 510006, China; 2111705055@e.gzhu.edu.cn (W.Y.); lhong@gzhu.edu.cn (H.L.); fpeng@gzhu.edu.cn (F.P.); gzdxlzl@gmail.com (Z.L.)
2. School of Chemistry and Chemical Engineering, South China University of Technology, Guangzhou 510640, China
3. School of Chemistry and Chemical Engineering, Wuhan University of Technology, Wuhan 430072, China; ljie@wit.edu.cn (J.L.)

* Correspondence: zqiao@gzhu.edu.cn

Received: 26 January 2019; Accepted: 17 March 2019; Published: date

Table of Contents

Lennard–Jones parameters of MOFs S2

Models of CH_4, N_2, H_2S, O_2, CO_2, H_2 and He S3

Lennard–Jones parameters and charges of adsorbates S4

Abbreviation list S4

Relationships between diffusivity *D* and MOF descriptors S5

Relationships between diffusion selectivities S_{diff} and MOF descriptors S8

Relationships between permeability *P* and MOF descriptors S13

Relationships between permselectivity S_{perm} and MOF descriptors S16

Relationships between permeability *P* and permselectivity S_{perm} S21

PC cover the ratio of variation information for 44 performance metrics S22

RMSE values and *R versus* four machine learnings S22

Predicted performance by DT, SVM, and BPNN *versus* simulated results S23

k times repeated *k*-fold cross-validation and principal component analysis S23

Principal component analysis S24

Decision tree S24

Random forest S25

Support vector machine S25

Back propagation neural network S25

Benchmark of permeability and permselectivity for 15 gas mixtures S26

Best CoRE-MOFMs for different gas mixtures S26

References S27

Table 1. Lennard–Jones parameters of metal–organic frameworks (MOFs) [1].

Atom	ε/k_B [K]	σ [Å]	Atom	ε/k_B [K]	σ [Å]	Atom	ε/k_B [K]	σ [Å]
Ac	16.60	3.10	Ge	190.69	3.81	Po	163.52	4.20
Ag	18.11	2.80	Gd	4.53	3.00	Pr	5.03	3.21
Al	254.09	4.01	H	22.14	2.57	Pt	40.25	2.45
Am	7.04	3.01	Hf	36.23	2.80	Pu	8.05	3.05
Ar	93.08	3.45	Hg	193.71	2.41	Ra	203.27	3.28
As	155.47	3.77	Ho	3.52	3.04	Rb	20.13	3.67
At	142.89	4.23	I	170.57	4.01	Re	33.21	2.63
Au	19.62	2.93	In	301.39	3.98	Rh	26.67	2.61
B	90.57	3.64	Ir	36.73	2.53	Rn	124.78	4.25
Ba	183.15	3.30	K	17.61	3.40	Ru	28.18	2.64
Be	42.77	2.45	Kr	110.69	3.69	S	137.86	3.59
Bi	260.63	3.89	La	8.55	3.14	Sb	225.91	3.94
Bk	6.54	2.97	Li	12.58	2.18	Sc	9.56	2.94
Br	126.29	3.73	Lu	20.63	3.24	Se	146.42	3.75
C	52.83	3.43	Lr	5.53	2.88	Si	202.27	3.83
Ca	119.75	3.03	Md	5.53	2.92	Sm	4.03	3.14
Cd	114.72	2.54	Mg	55.85	2.69	Sn	285.28	3.91
Ce	6.54	3.17	Mn	6.54	2.64	Sr	118.24	3.24
Cf	6.54	2.95	Mo	28.18	2.72	Ta	40.75	2.82
Cl	114.21	3.52	N	34.72	3.26	Tb	3.52	3.07
Cm	6.54	2.96	Na	15.09	2.66	Tc	24.15	2.67
Co	7.04	2.56	Ne	21.13	2.66	Te	200.25	3.98
Cr	7.55	2.69	Nb	29.69	2.82	Th	13.08	3.03
Cu	2.52	3.11	Nd	5.03	3.18	Ti	8.55	2.83
Cs	22.64	4.02	No	5.53	2.89	TI	342.14	3.87
Dy	3.52	3.05	Ni	7.55	2.52	Tm	3.02	3.01
Eu	4.03	3.11	Np	9.56	3.05	U	11.07	3.02
Er	3.52	3.02	O	30.19	3.12	V	8.05	2.80
Es	6.04	2.94	Os	18.62	2.78	W	33.71	2.73
F	25.16	3.00	P	153.46	3.69	Xe	167.04	3.92
Fe	6.54	2.59	Pa	11.07	3.05	Y	36.23	2.98
Fm	6.04	2.93	Pb	333.59	3.83	Yb	114.72	2.99
Fr	25.16	4.37	Pd	24.15	2.58	Zn	62.39	2.46
Ga	208.81	3.90	Pm	4.53	3.16	Zr	34.72	2.78

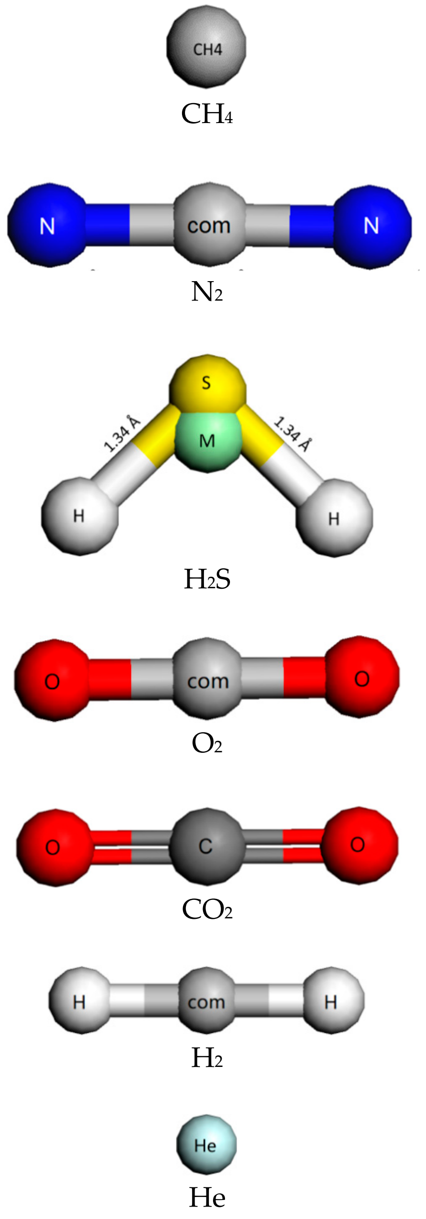

Figure S1. Models of CH_4, N_2, H_2S, O_2, CO_2, H_2, and He [2].

Table S2. Lennard–Jones parameters and charges of adsorbates [3].

Atom	ε/k_B [K]	σ [Å]	Charge (e)	Atom	ε/k_B [K]	σ [Å]	Charge (e)
C_CO_2	27.0	2.80	+0.700	H_H_2S	50.0	2.50	+0.210
O_CO_2	79.0	3.05	–0.350	S_H_2S	122.0	3.60	0
CH_4	148.0	3.73	0	M_H_2S	0	0	–0.420
N_N_2	36.0	3.31	–0.482	H_H_2	0	0	+0.468
com_N_2	0	0	+0.964	com_H_2	36.7	2.96	–0.936
O_O_2	49.0	3.02	–0.113	He	10.9	2.64	0
com _O_2	0	0	+0.226				

Abbreviation list:

Full name	Abbreviation	Full name	Abbreviation
Computation-ready, experimental metal–organic framework membranes	CoRE-MOFMs	Monte Carlo	MC
Molecular dynamics	MD	Electrostatic potential-optimized charge scheme	MEPO-Qeq
Universal force field	UFF	Pore limiting diameter	PLD
Large cavity diameter	LCD	Volumetric surface area	VSA
Pore size distribution	PSD	Principal component analysis	PCA
Decision tree	DT	Random forest	RF
Support vector machine	SVM	Back propagation neural network	BPNN
Root mean square error	RMSE	Principal component	PC

Name interpretation	Abbreviation	Name interpretation	Abbreviation
Number of replication in k times k-fold cross-validation	k	The linear correlation values	R
Porosity	ϕ	Density	ρ
Permeability	P	Diffusion coefficient	D
Permselectivity	S_{perm}	Diffusion selectivity	S_{diff}
Henry's constant	K_i		

Relationships between diffusivity D and MOF descriptors

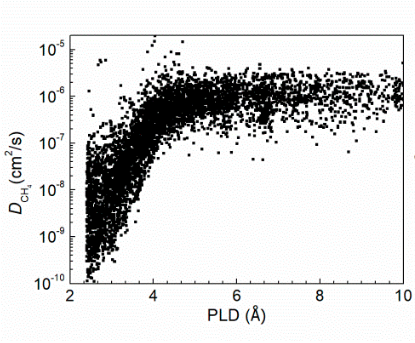

(**a1**) D_{CH_4} ~ PLD

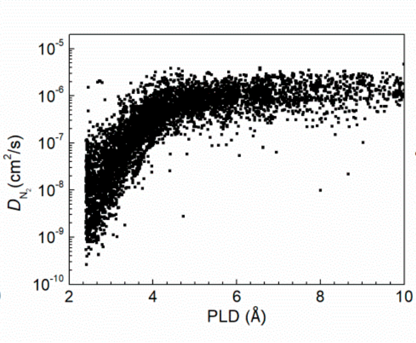

(**a2**) D_{N_2} ~ PLD

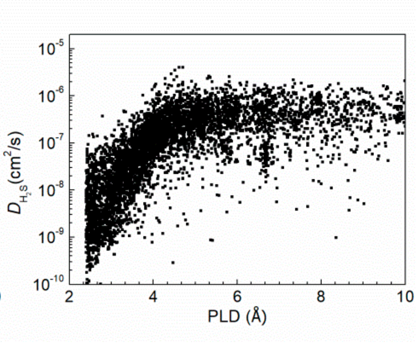

(**a3**) D_{H_2S} ~ PLD

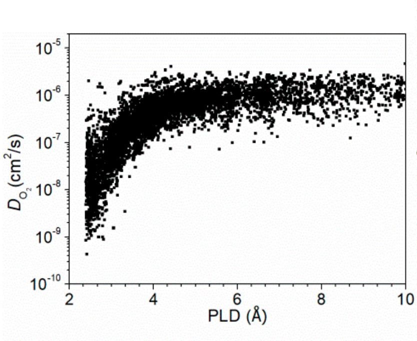

(**a4**) D_{O_2} ~ PLD

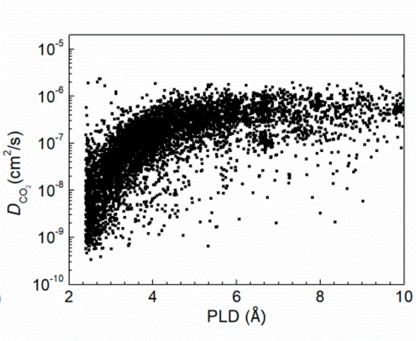

(**a5**) D_{CO_2} ~ PLD

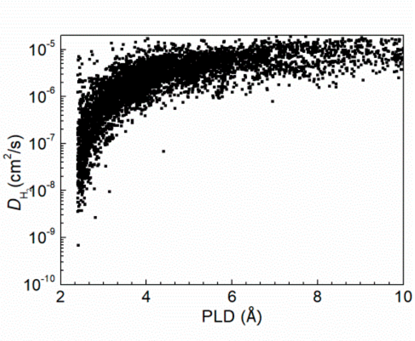

(**a6**) D_{H_2} ~ PLD

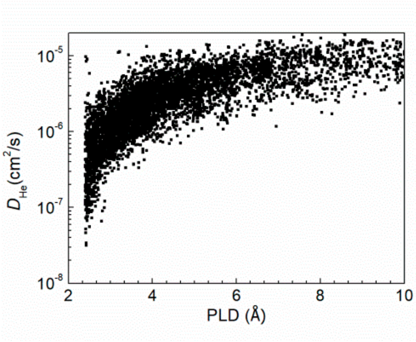

(**a7**) D_{He} ~ PLD

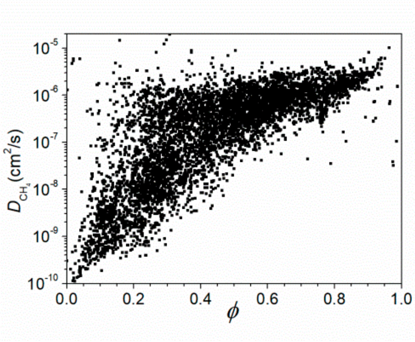

(**b1**) D_{CH_4} ~

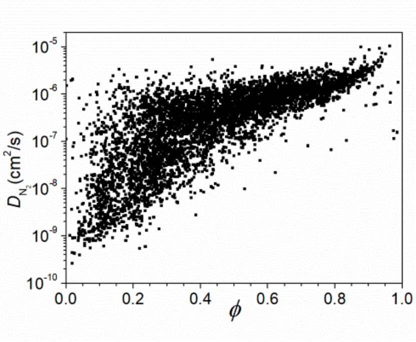

(**b2**) D_{N_2} ~

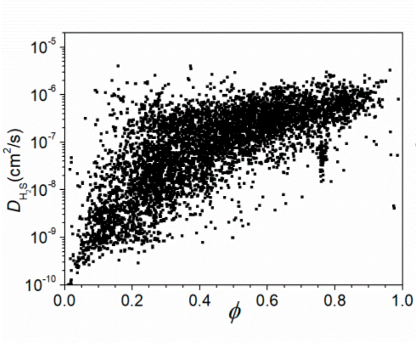

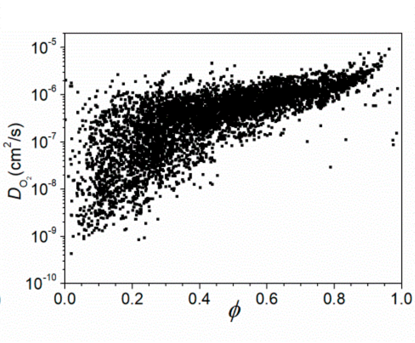

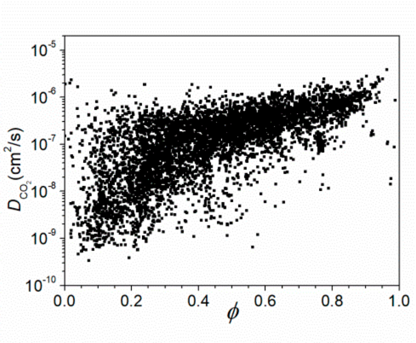

(**b3**) D_{H_2S} ~ (**b4**) D_{O_2} ~ (**b5**) D_{CO_2} ~

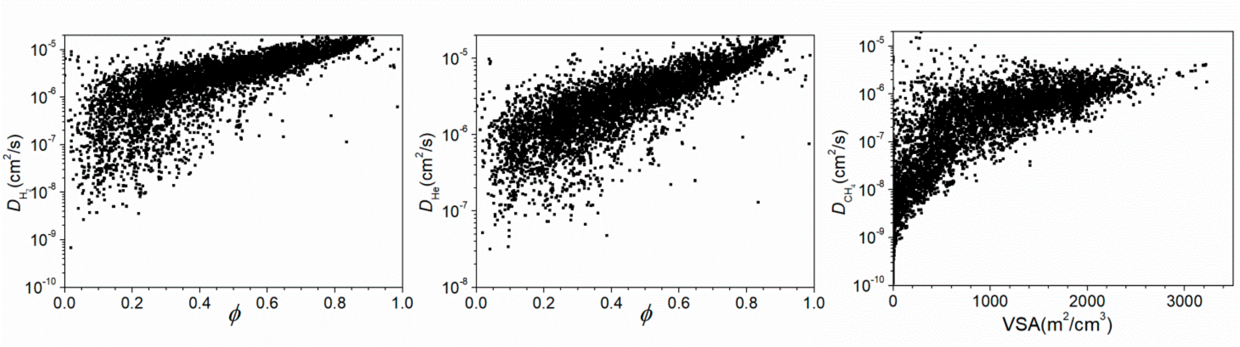

(**b6**) D_{H_2} ~ (**b7**) D_{He} ~ (**c1**) D_{CH_4} ~ VSA

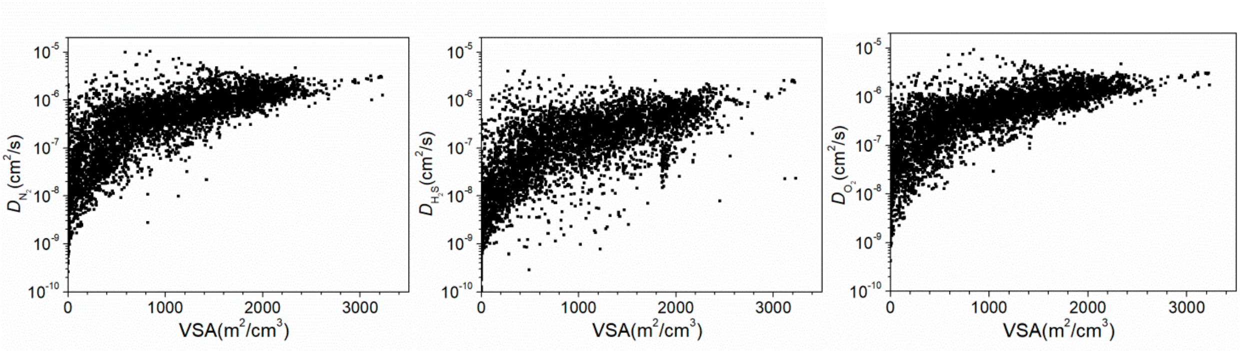

(**c2**) D_{N_2} ~ VSA (**c3**) D_{H_2S} ~ VSA (**c4**) D_{O_2} ~ VSA

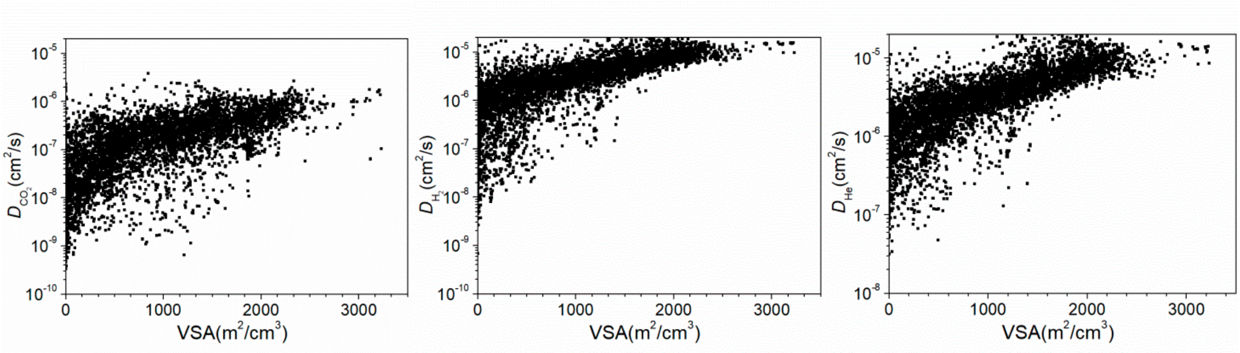

(**c5**) D_{CO_2} ~ VSA (**c6**) D_{H_2} ~ VSA (**c7**) D_{He} ~ VSA

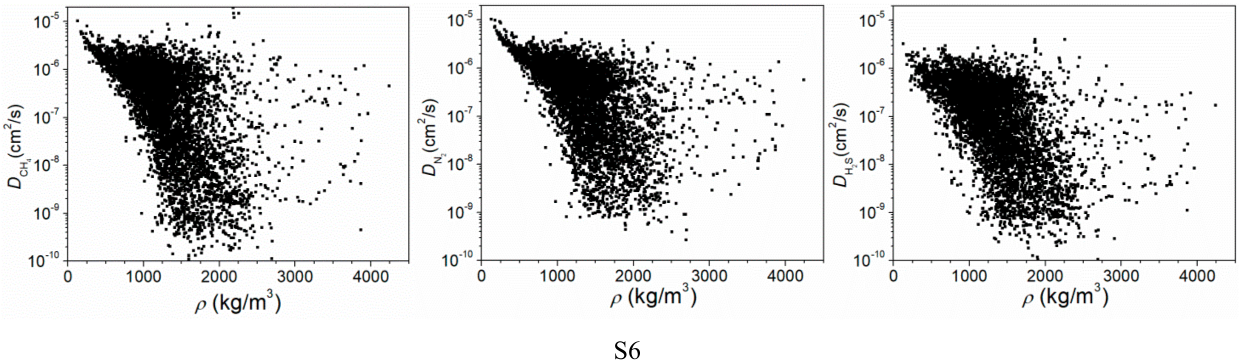

(**d1**) D_{CH_4} ~ (**d2**) D_{N_2} ~ (**d3**) D_{H_2S} ~

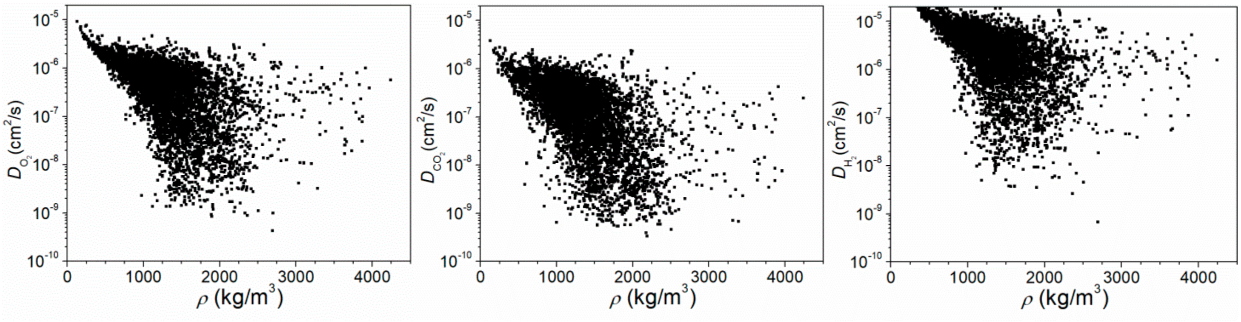

(**d4**) D_{O_2} ~ (**d5**) D_{CO_2} ~ (**d6**) D_{H_2} ~

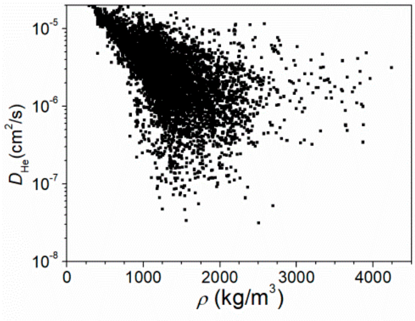

(**d7**) D_{He} ~

Figure S2. Relationships between diffusivity *D* and MOF descriptors: (**a**) *D* and PLD, (**b**) *D* and , (**c**) *D* and VSA, (**d**) *D* and, (1–7) represent different gases (CH_4, N_2, H_2S, O_2, CO_2, H_2, and He).

Relationships between diffusion selectivities S_{diff} and MOF descriptors

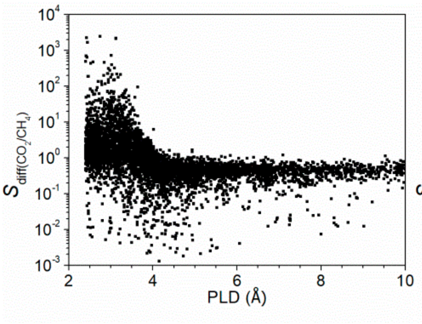

(**a1**) $S_{diff(CO_2/CH_4)}$ ~ PLD

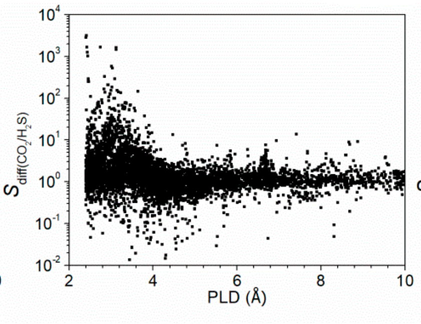

(**a2**) $S_{diff(CO_2/H_2S)}$ ~ PLD

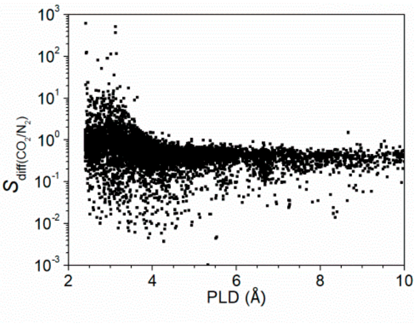

(**a3**) $S_{diff(CO_2/N_2)}$ ~ PLD

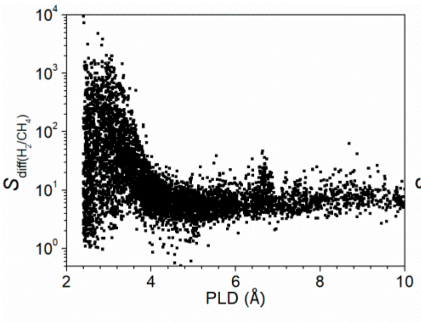

(**a4**) $S_{diff(H_2/CH_4)}$ ~ PLD

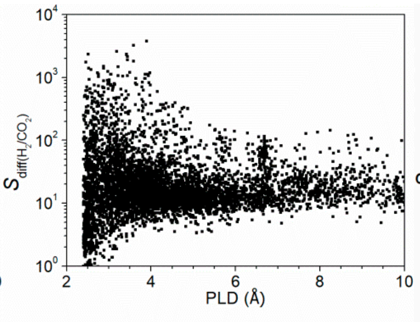

(**a5**) $S_{diff(H_2/CO_2)}$ ~ PLD

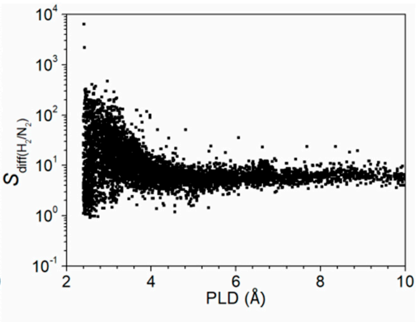

(**a6**) $S_{diff(H_2/N_2)}$ ~ PLD

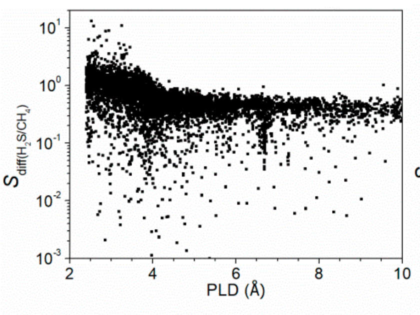

(**a7**) $S_{diff(H_2S/CH_4)}$ ~ PLD

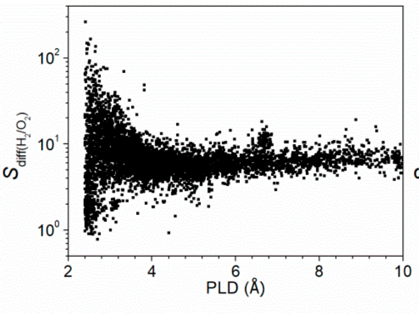

(**a8**) $S_{diff(H_2/O_2)}$ ~ PLD

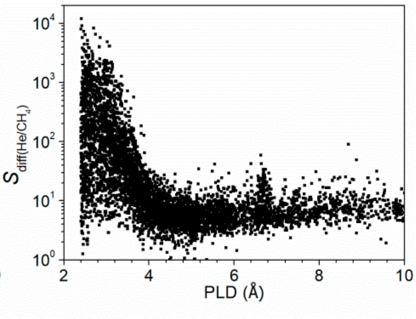

(**a9**) $S_{diff(He/CH_4)}$ ~ PLD

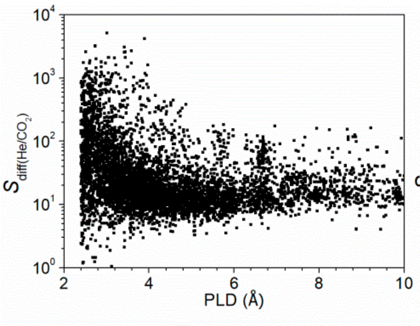

(**a10**) $S_{diff(He/CO_2)}$ ~ PLD

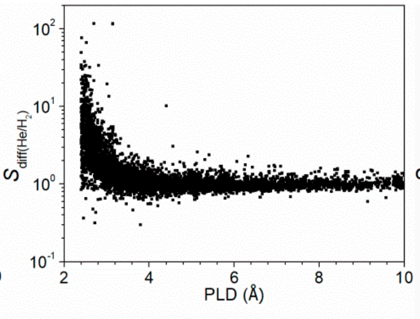

(**a11**) $S_{diff(He/H_2)}$ ~ PLD

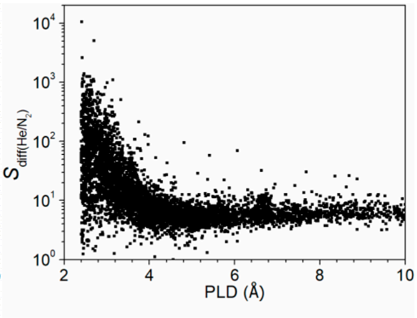

(**a12**) $S_{diff(He/N_2)}$ ~ PLD

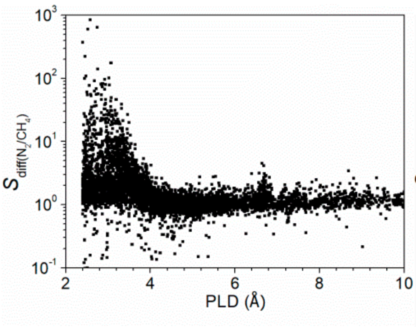

(a13) $S_{diff(N_2/CH_4)}$ ~ PLD

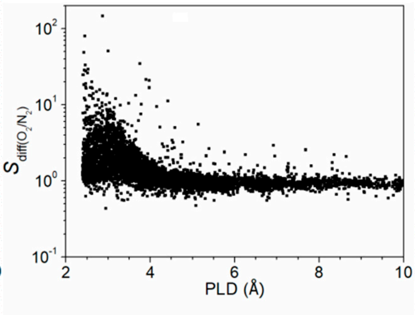

(a14) $S_{diff(O_2/N_2)}$ ~ PLD

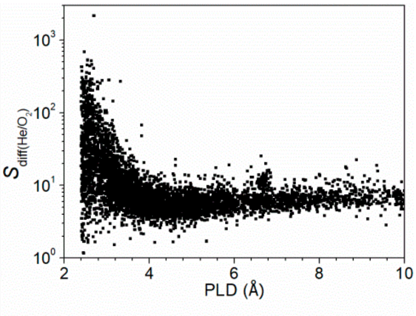

(a15) $S_{diff(He/O_2)}$ ~ PLD

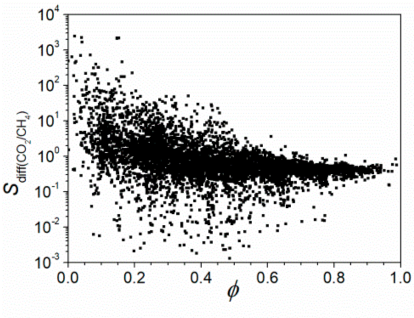

(b1) $S_{diff(CO_2/CH_4)}$ ~

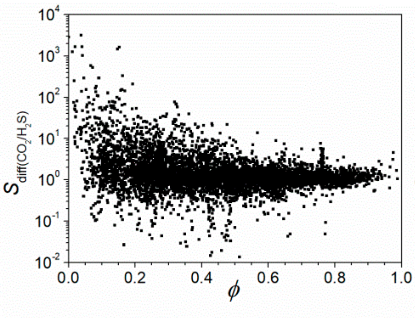

(b2) $S_{diff(CO_2/H_2S)}$ ~

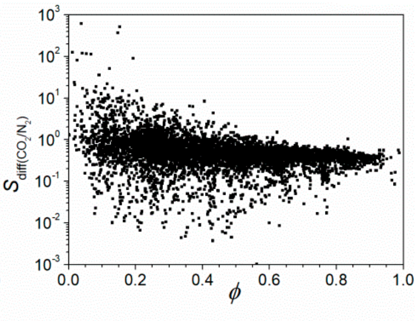

(b3) $S_{diff(CO_2/N_2)}$ ~

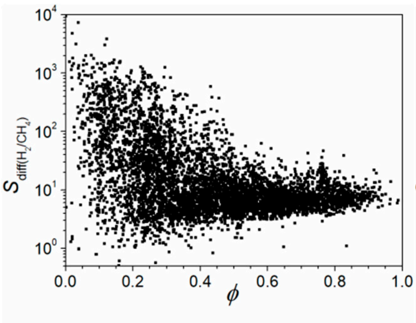

(b4) $S_{diff(H_2/CH_4)}$ ~

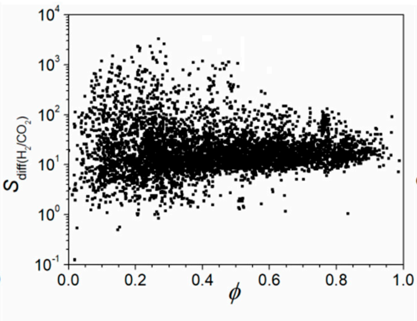

(b5) $S_{diff(H_2/CO_2)}$ ~

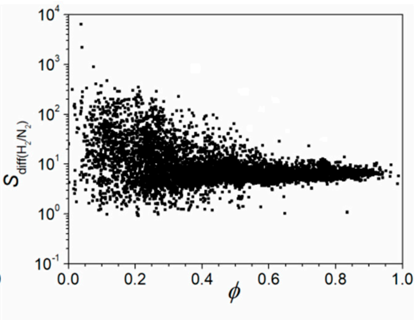

(b6) $S_{diff(H_2/N_2)}$ ~

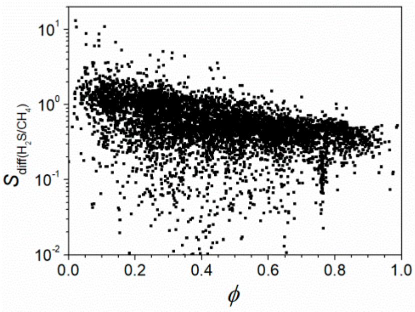

(b7) $S_{diff(H_2S/CH_4)}$ ~

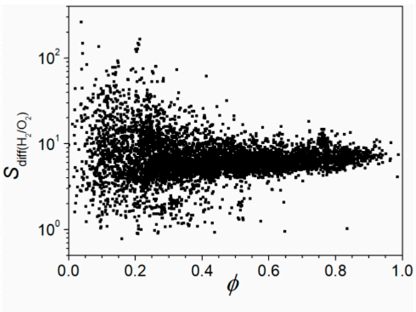

(b8) $S_{diff(H_2/O_2)}$ ~

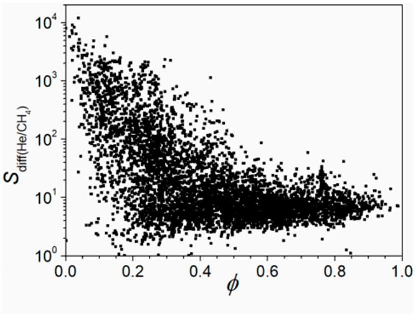

(b9) $S_{diff(He/CH_4)}$ ~

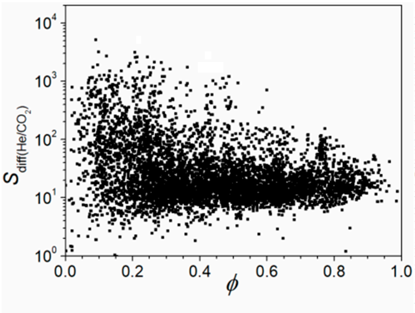

(**b10**) $S_{diff(He/CO_2)}$ ~

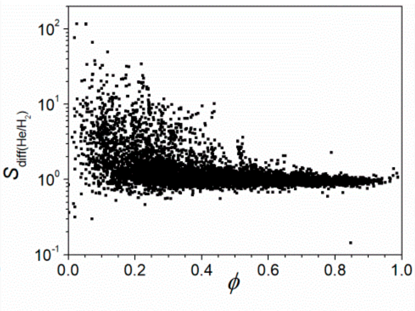

(**b11**) $S_{diff(He/H_2)}$ ~

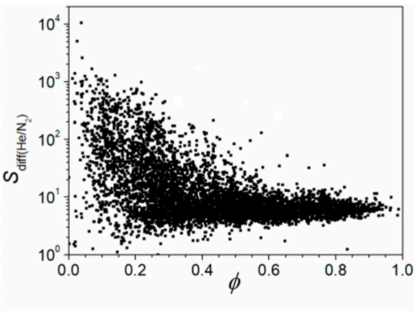

(**b12**) $S_{diff(He/N_2)}$ ~

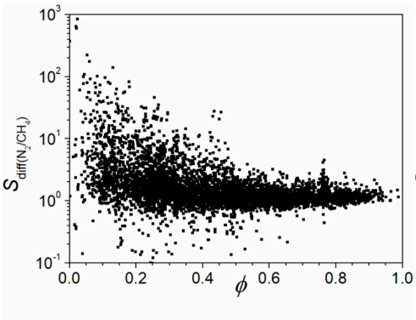

(**b13**) $S_{diff(N_2/CH_4)}$ ~

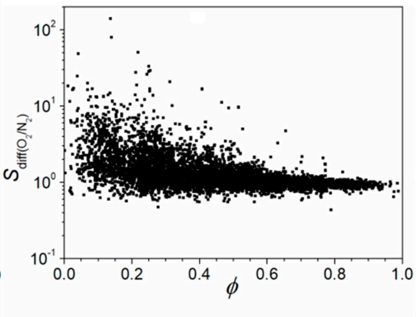

(**b14**) $S_{diff(O_2/N_2)}$ ~

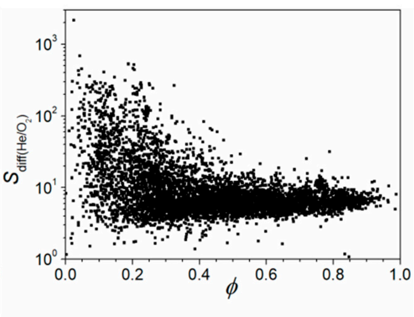

(**b15**) $S_{diff(He/O_2)}$ ~

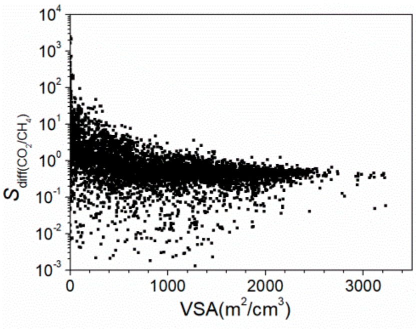

(**c1**) $S_{diff(CO_2/CH_4)}$ ~ VSA

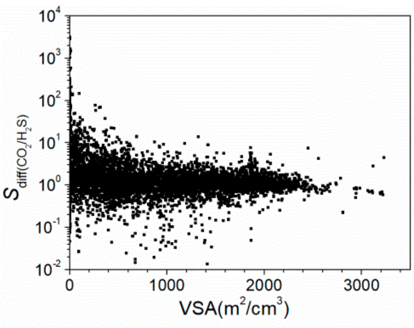

(**c2**) $S_{diff(CO_2/H_2S)}$ ~ VSA

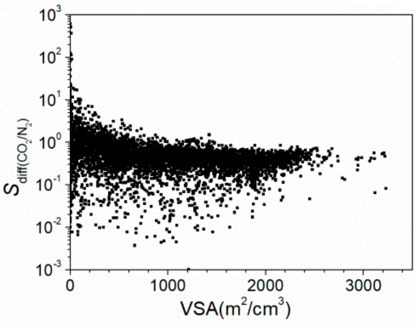

(**c3**) $S_{diff(CO_2/N_2)}$ ~ VSA

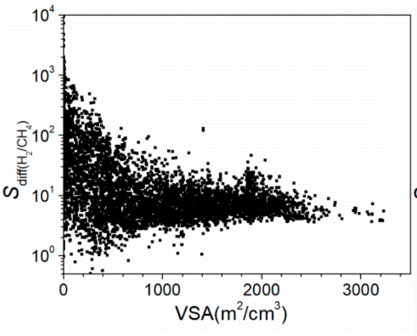

(**c4**) $S_{diff(H_2/CH_4)}$ ~ VSA

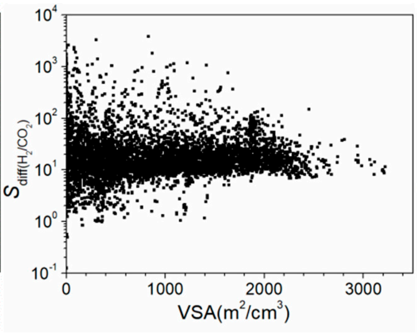

(**c5**) $S_{diff(H_2/CO_2)}$ ~ VSA

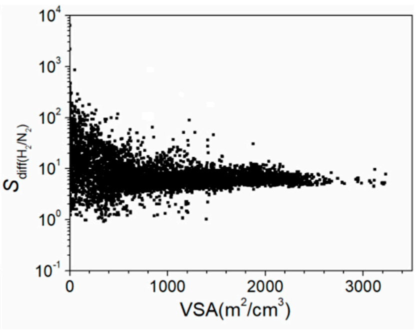

(**c6**) $S_{diff(H_2/N_2)}$ ~ VSA

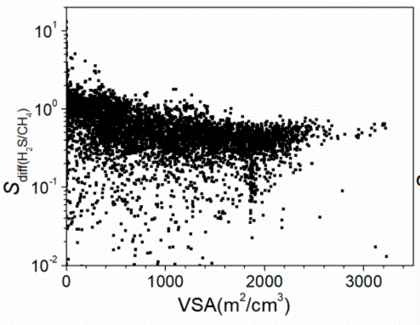

(**c7**) $S_{diff(H_2S/CH_4)}$ ~ VSA

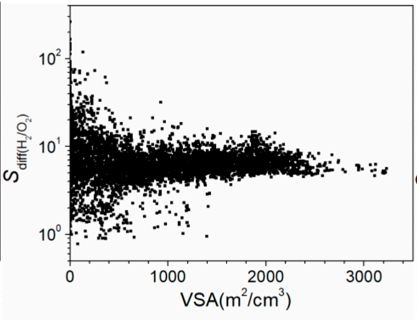

(**c8**) $S_{diff(H_2/O_2)}$ ~ VSA

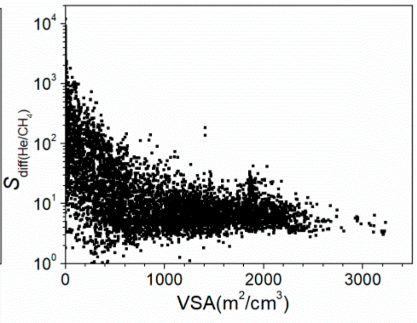

(**c9**) $S_{diff(He/CH_4)}$ ~ VSA

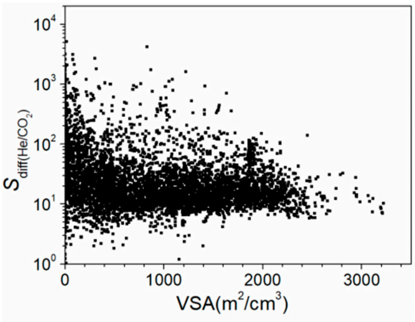

(**c10**) $S_{diff(He/CO_2)}$ ~ VSA

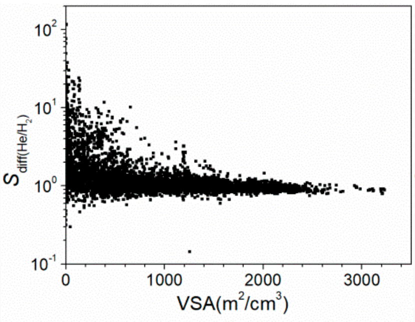

(**c11**) $S_{diff(He/H_2)}$ ~ VSA

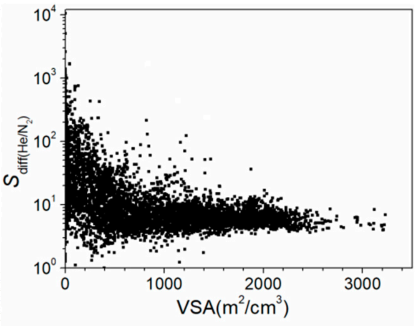

(**c12**) $S_{diff(He/N_2)}$ ~ VSA

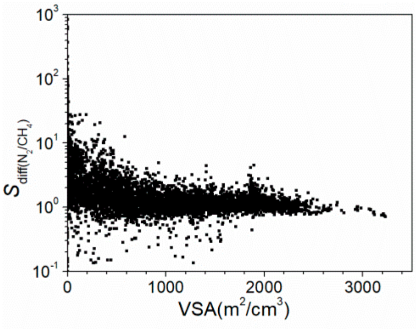

(**c13**) $S_{diff(N_2/CH_4)}$ ~ VSA

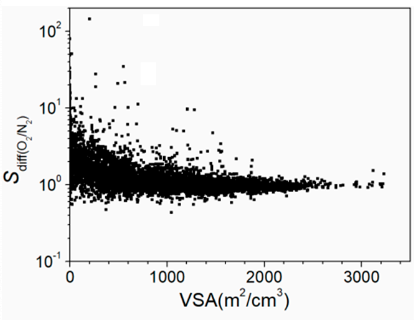

(**c14**) $S_{diff(O_2/N_2)}$ ~ VSA

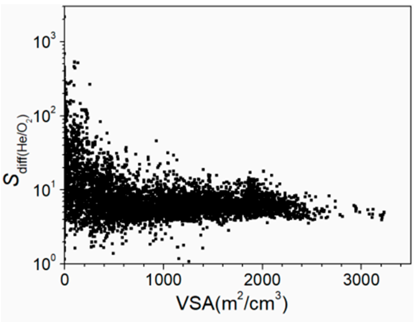

(**c15**) $S_{diff(He/O_2)}$ ~ VSA

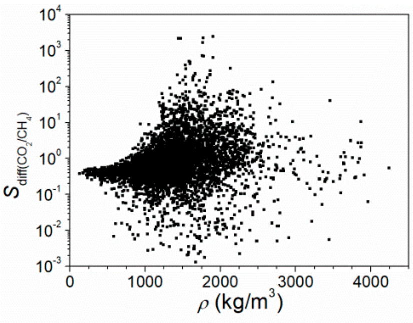

(**d1**) $S_{diff(CO_2/CH_4)}$ ~

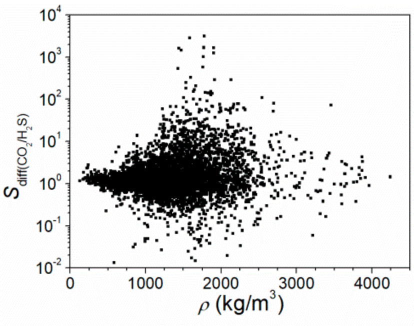

(**d2**) $S_{diff(CO_2/H_2S)}$ ~

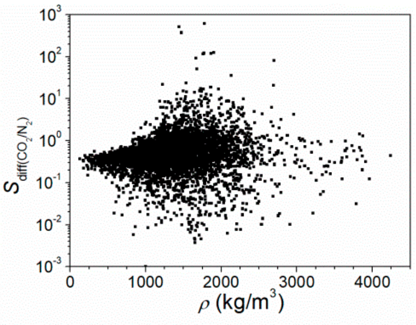

(**d3**) $S_{diff(CO_2/N_2)}$ ~

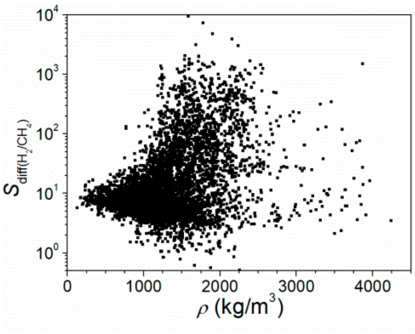

(**d4**) $S_{diff(H_2/CH_4)}$ ~

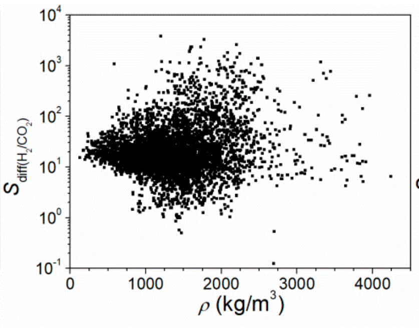

(**d5**) $S_{diff(H_2/CO_2)}$ ~

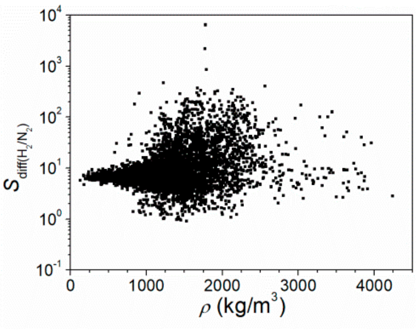

(**d6**) $S_{diff(H_2/N_2)}$ ~

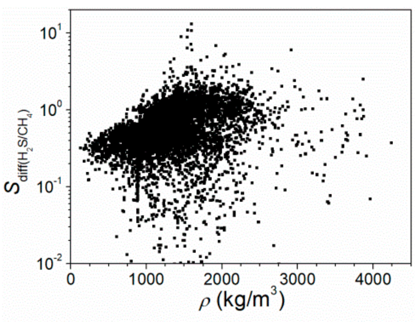

(**d7**) $S_{diff(H_2S/CH_4)}$ ~

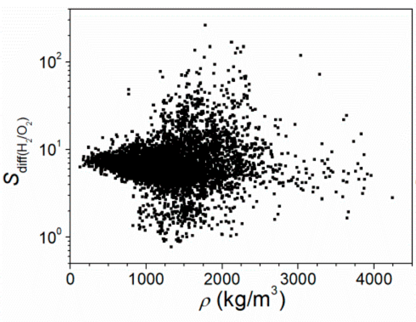

(**d8**) $S_{diff(H_2/O_2)}$ ~

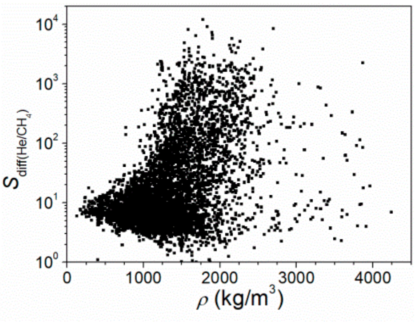

(**d9**) $S_{diff(He/CH_4)}$ ~

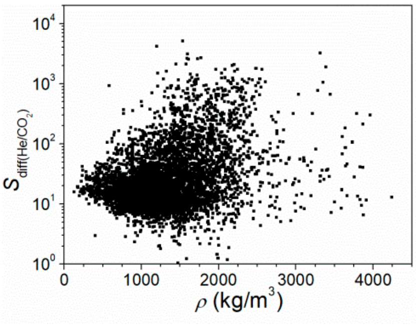

(**d10**) $S_{diff(He/CO_2)}$ ~

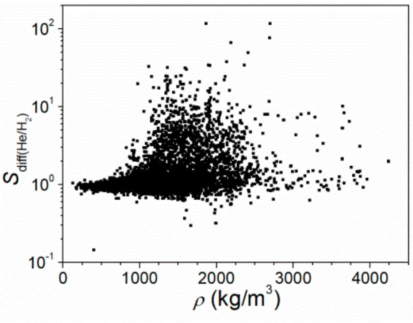

(**d11**) $S_{diff(He/H_2)}$ ~

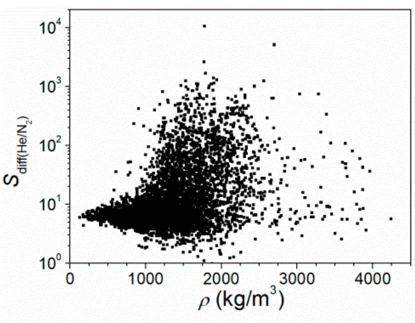

(**d12**) $S_{diff(He/N_2)}$ ~

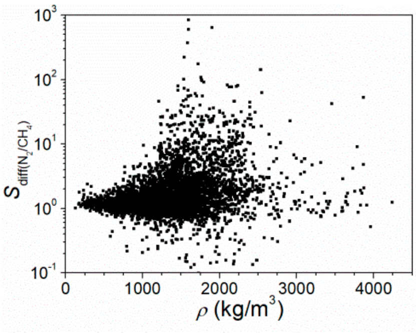

(**d13**) $S_{diff(N_2/CH_4)}$ ~

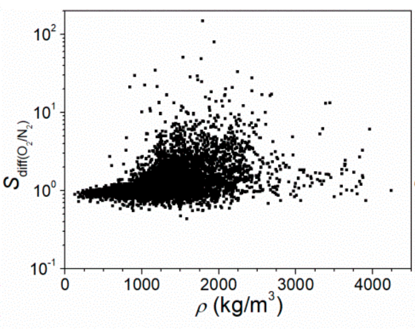

(**d14**) $S_{diff(O_2/N_2)}$ ~

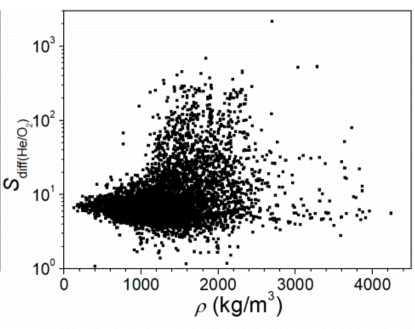

(**d15**) $S_{diff(He/O_2)}$ ~

Figure S3. Relationships between diffusion selectivities S_{diff} and MOF descriptors: (**a**) S_{diff} and PLD, (**b**) S_{diff} and (**c**) S_{diff} and VSA, (**d**) S_{diff} and , (1–15) represent 15 different gas mixtures (CO_2/CH_4, CO_2/H_2S, CO_2/N_2, H_2/CH_4, H_2/CO_2, H_2/N_2, H_2S/CH_4, H_2/O_2, He/CH_4, He/CO_2, He/H_2, He/N_2, N_2/CH_4, O_2/N_2, and He/O_2).

Relationships between permeability *P* and MOF descriptors

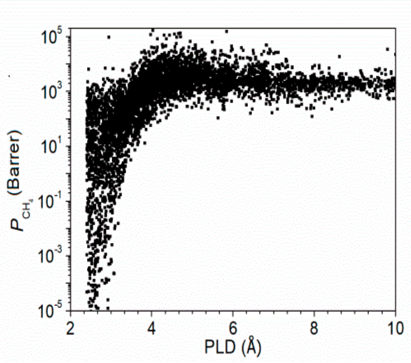

(**a1**) P_{CH_4} ~ PLD

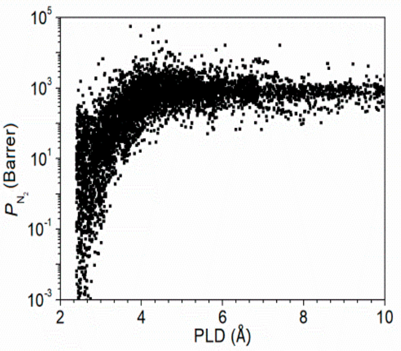

(**a2**) P_{N_2} ~ PLD

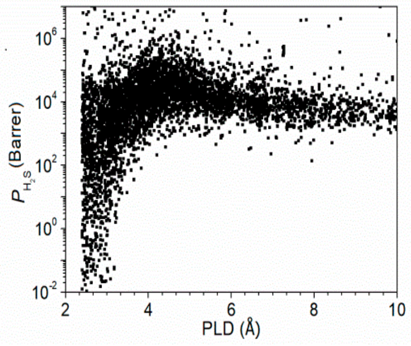

(**a3**) P_{H_2S} ~ PLD

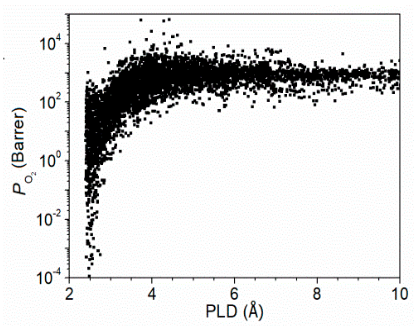

(**a4**) P_{O_2} ~ PLD

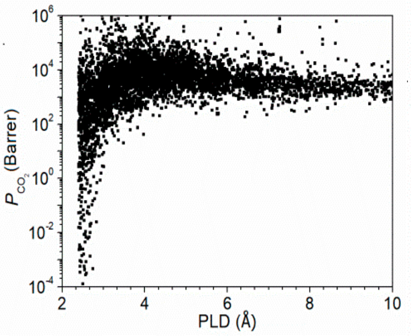

(**a5**) P_{CO_2} ~ PLD

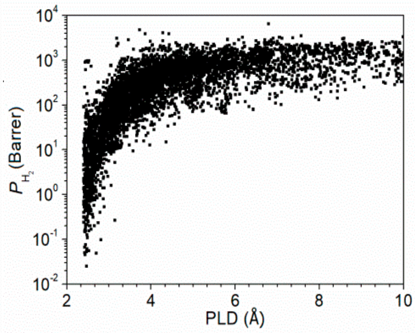

(**a6**) P_{H_2} ~ PLD

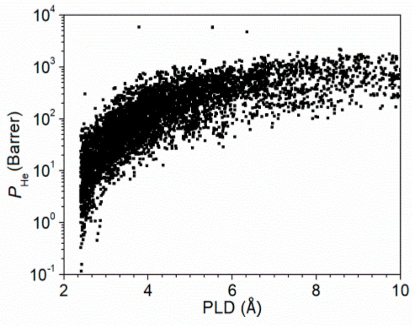

(**a7**) P_{He} ~ PLD

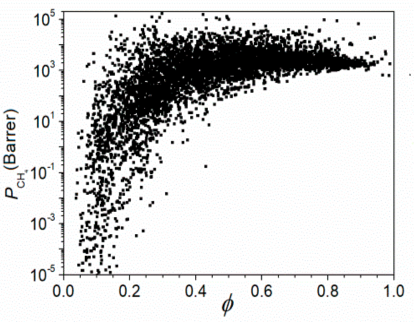

(**b1**) P_{CH_4} ~

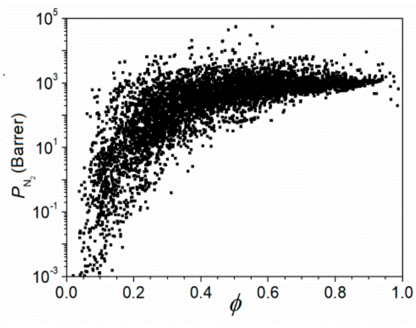

(**b2**) P_{N_2} ~

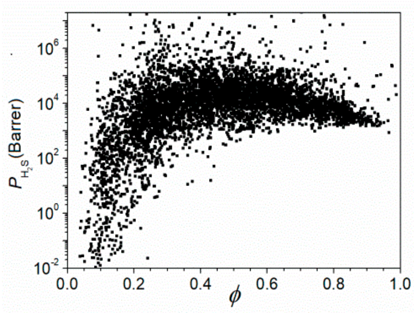

(**b3**) P_{H_2S} ~

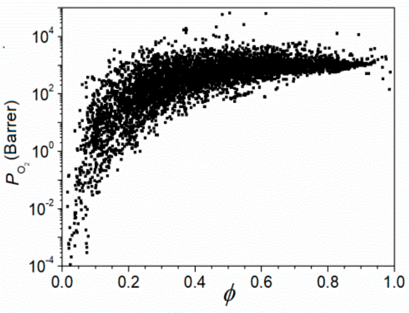

(**b4**) P_{O_2} ~

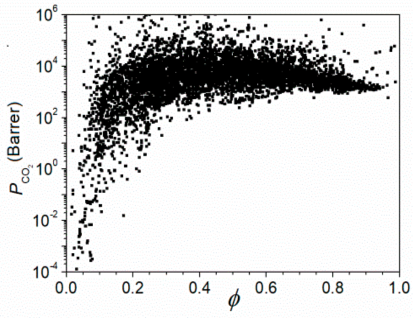

(**b5**) P_{CO_2} ~

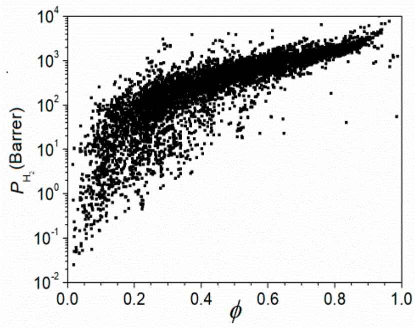

(**b6**) P_{H_2} ~

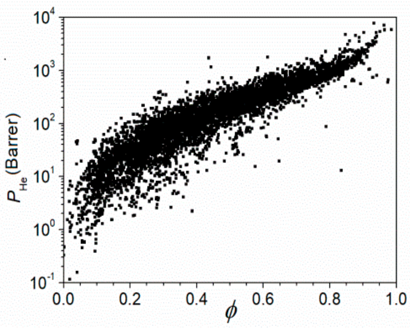

(**b7**) P_{He} ~

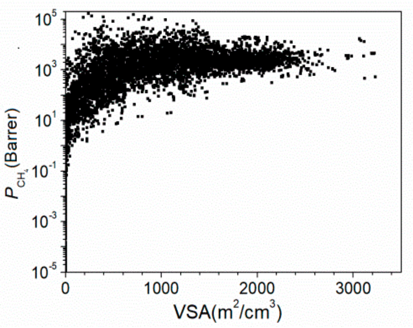

(**c1**) P_{CH_4} ~ VSA

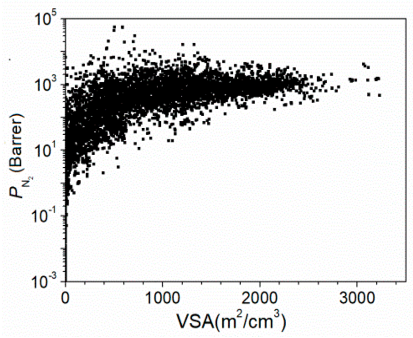

(**c2**) P_{N_2} ~ VSA

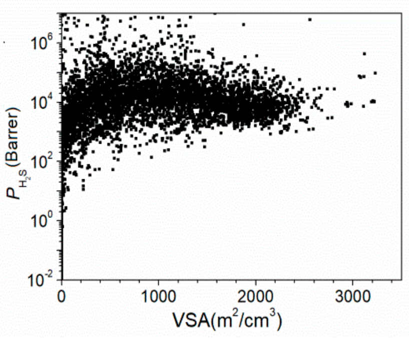

(**c3**) P_{H_2S} ~ VSA

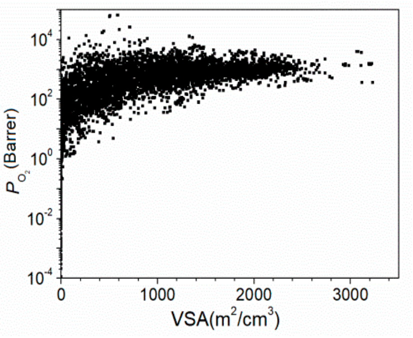

(**c4**) P_{O_2} ~ VSA

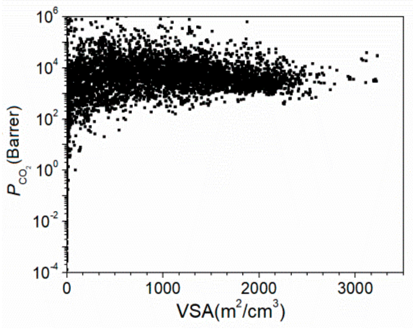

(**c5**) P_{CO_2} ~ VSA

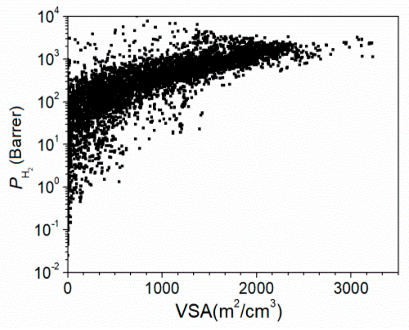

(**c6**) P_{H_2} ~ VSA

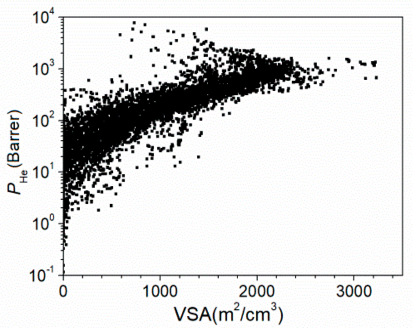

(**c7**) P_{He} ~ VSA

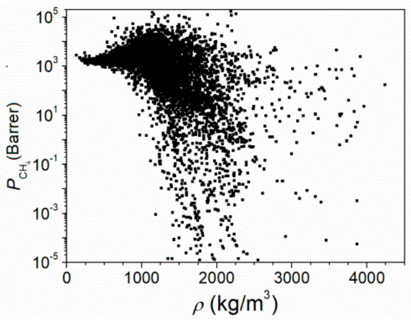

(**d1**) P_{CH_4} ~

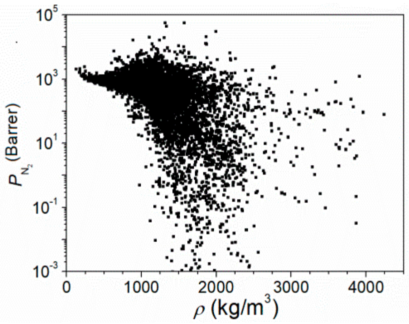

(**d2**) P_{N_2} ~

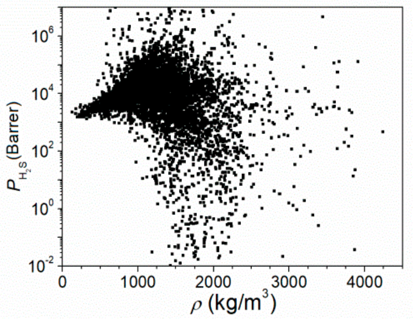

(**d3**) P_{H_2S} ~

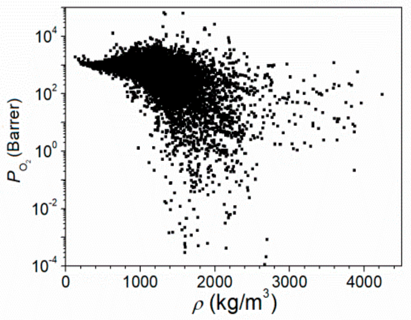

(**d4**) P_{O_2} ~

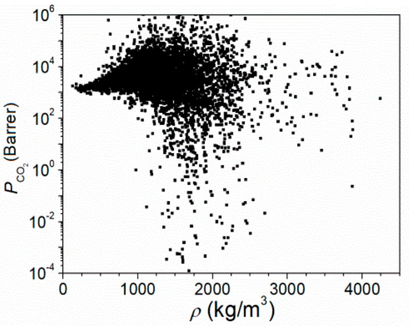

(**d5**) P_{CO_2} ~

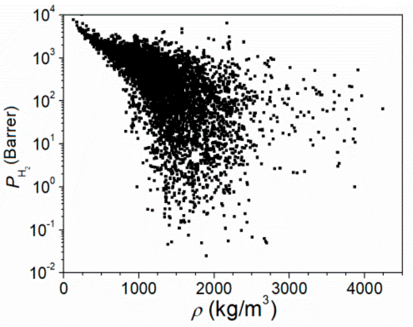

(**d6**) P_{H_2} ~

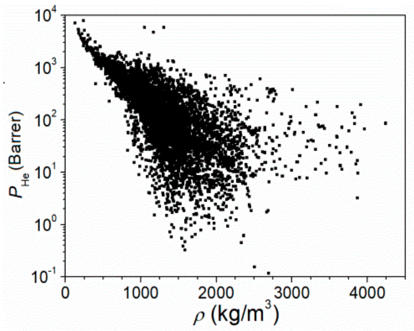

(**d7**) P_{He} ~

Figure S4. Relationships between permeability *P* and MOF descriptors: (**a**) *P* and PLD, (**b**) *P* and, (**c**) *P* and VSA, (**f**) *P* and, (1–7) represent different gases (CH_4, N_2, H_2S, O_2, CO_2, H_2, and He).

Relationships between permselectivity S_{perm} and MOF descriptors

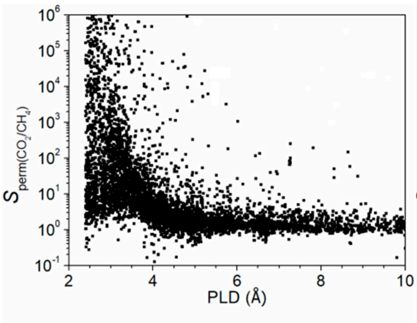

(**a1**) $S_{perm(CO_2/CH_4)}$ ~ PLD

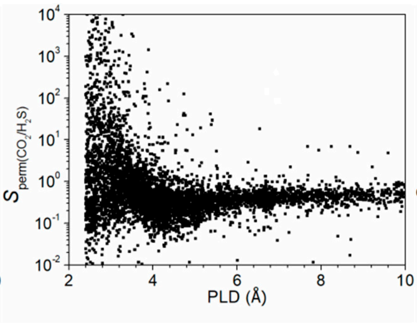

(**a2**) $S_{perm(CO_2/H_2S)}$ ~ PLD

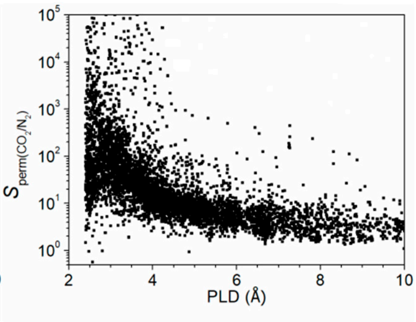

(**a3**) $S_{perm(CO_2/N_2)}$ ~ PLD

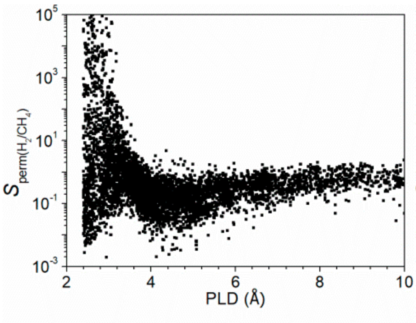

(**a4**) $S_{perm(H_2/CH_4)}$ ~ PLD

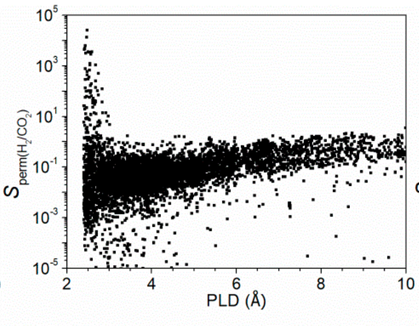

(**a5**) $S_{perm(H_2/CO_2)}$ ~ PLD

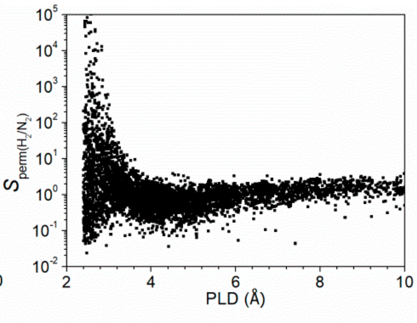

(**a6**) $S_{perm(H_2/N_2)}$ ~ PLD

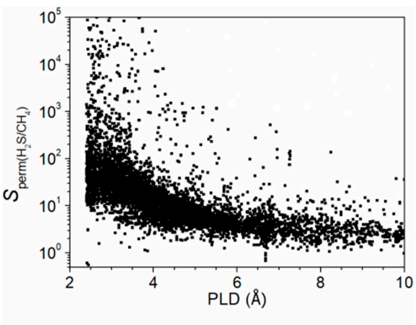

(**a7**) $S_{perm(H_2S/CH_4)}$ ~ PLD

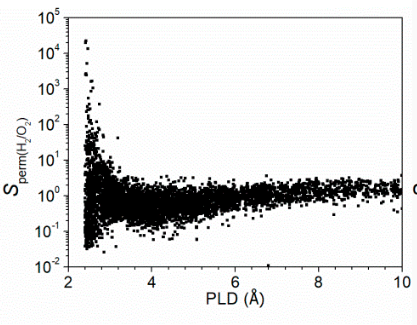

(**a8**) $S_{perm(H_2/O_2)}$ ~ PLD

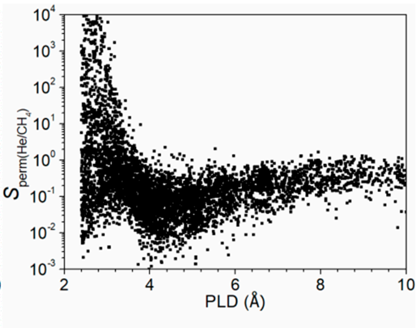

(**a9**) $S_{perm(He/CH_4)}$ ~ PLD

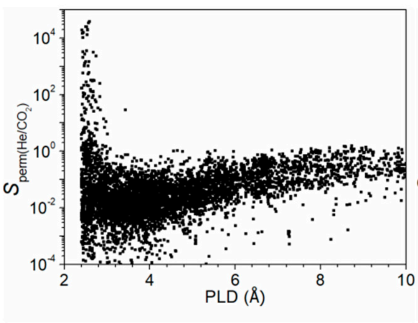

(**a10**) $S_{perm(He/CO_2)}$ ~ PLD

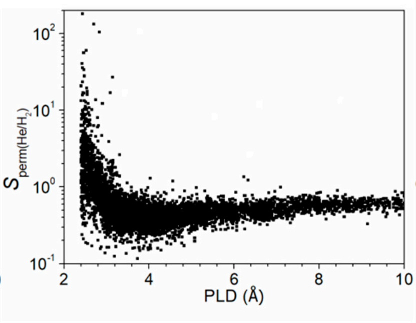

(**a11**) $S_{perm(He/H_2)}$ ~ PLD

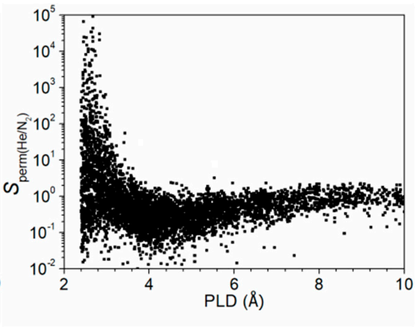

(**a12**) $S_{perm(He/N_2)}$ ~ PLD

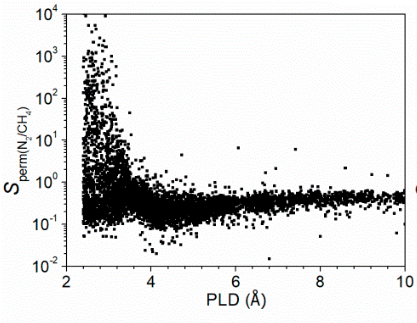

(**a13**) $S_{\mathrm{perm(N_2/CH_4)}}$ ~ PLD

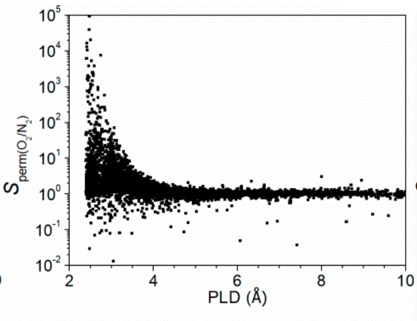

(**a14**) $S_{\mathrm{perm(O_2/N_2)}}$ ~ PLD

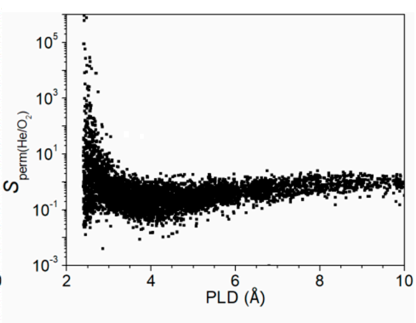

(**a15**) $S_{\mathrm{perm(He/O_2)}}$ ~ PLD

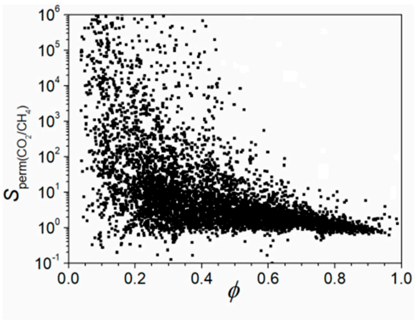

(**b1**) $S_{\mathrm{perm(CO_2/CH_4)}}$ ~

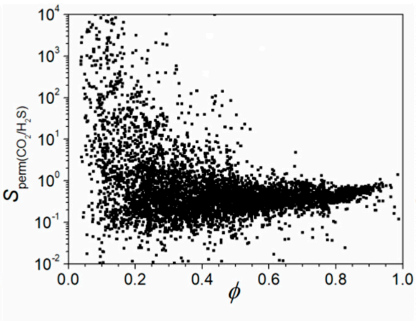

(**b2**) $S_{\mathrm{perm(CO_2/H_2S)}}$ ~

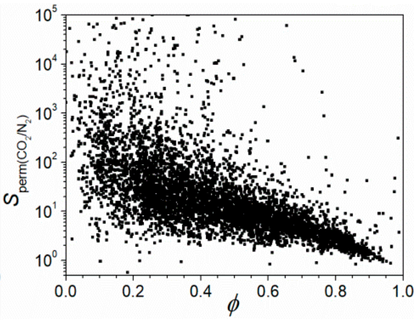

(**b3**) $S_{\mathrm{perm(CO_2/N_2)}}$ ~

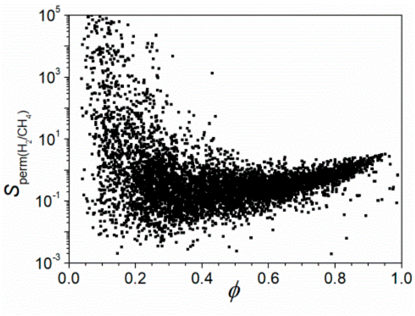

(**b4**) $S_{\mathrm{perm(H_2/CH_4)}}$ ~

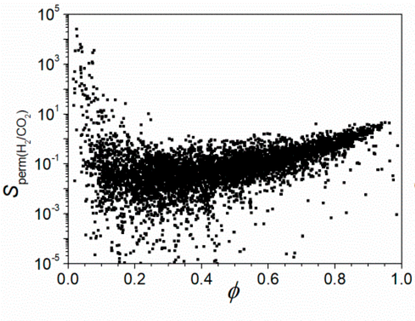

(**b5**) $S_{\mathrm{perm(H_2/CO_2)}}$ ~

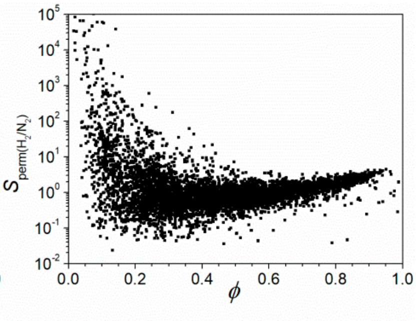

(**b6**) $S_{\mathrm{perm(H_2/N_2)}}$ ~

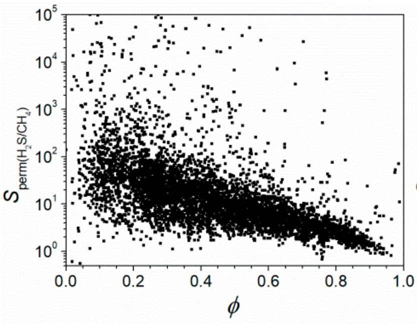

(**b7**) $S_{\mathrm{perm(H_2S/CH_4)}}$ ~

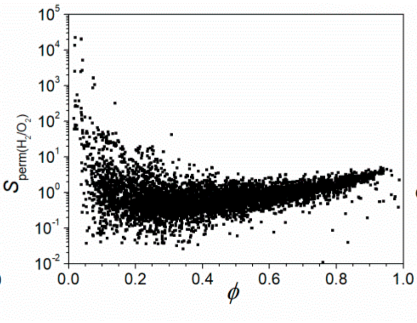

(**b8**) $S_{\mathrm{perm(H_2/O_2)}}$ ~

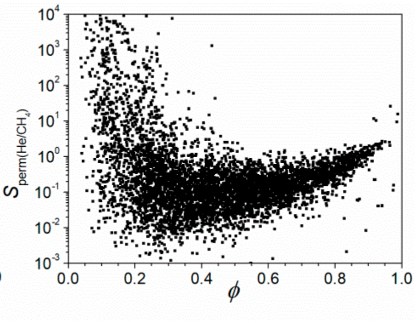

(**b9**) $S_{\mathrm{perm(He/CH_4)}}$ ~

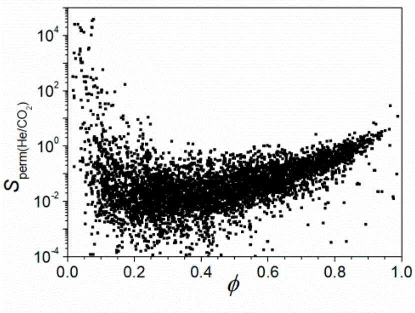

(**b10**) $S_{perm(He/CO_2)}$ ~

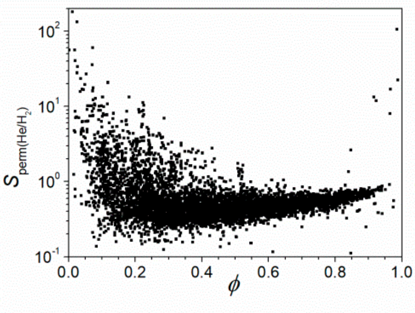

(**b11**) $S_{perm(He/H_2)}$ ~

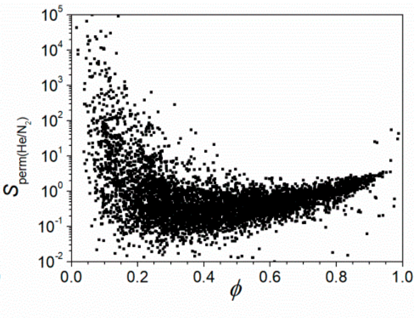

(**b12**) $S_{perm(He/N_2)}$ ~

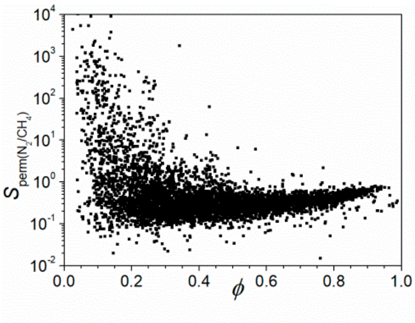

(**b13**) $S_{perm(N_2/CH_4)}$ ~

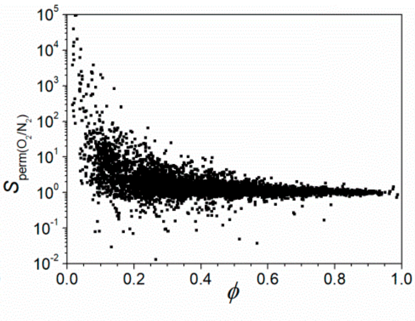

(**b14**) $S_{perm(O_2/N_2)}$ ~

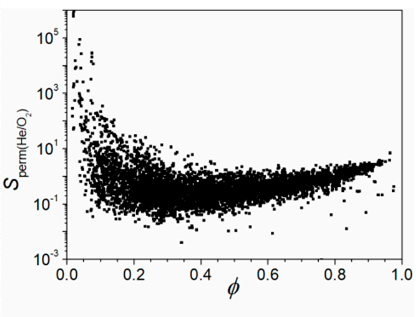

(**b15**) $S_{perm(He/O_2)}$ ~

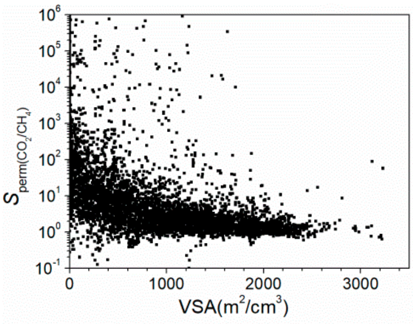

(**c1**) $S_{perm(CO_2/CH_4)}$ ~ VSA

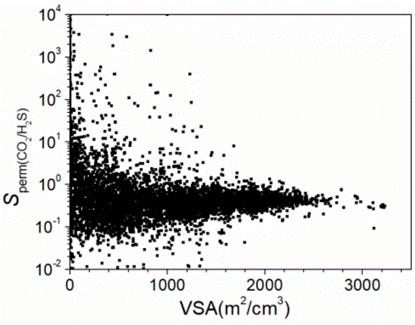

(**c2**) $S_{perm(CO_2/H_2S)}$ ~ VSA

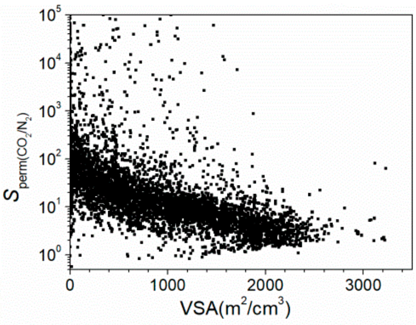

(**c3**) $S_{perm(CO_2/N_2)}$ ~ VSA

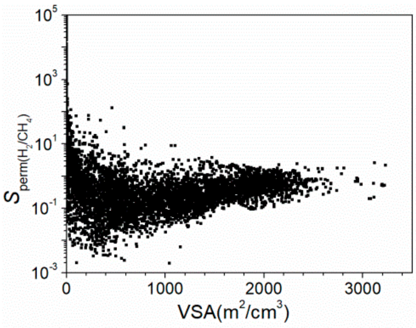

(**c4**) $S_{perm(H_2/CH_4)}$ ~ VSA

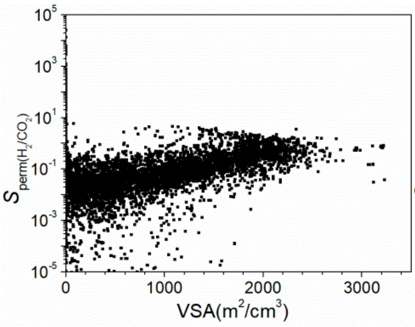

(**c5**) $S_{perm(H_2/CO_2)}$ ~ VSA

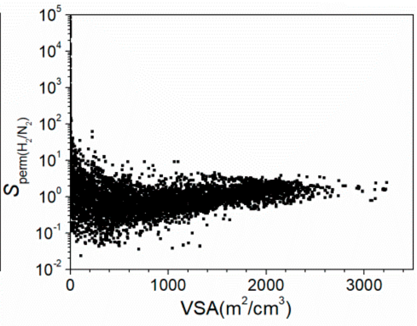

(**c6**) $S_{perm(H_2/N_2)}$ ~ VSA

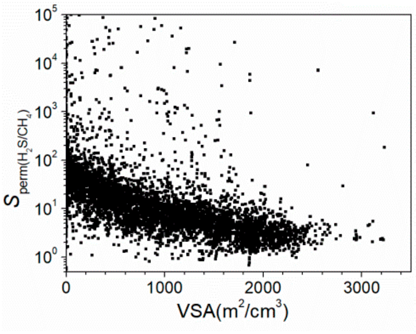

(c7) $S_{perm(H_2S/CH_4)}$ ~ VSA

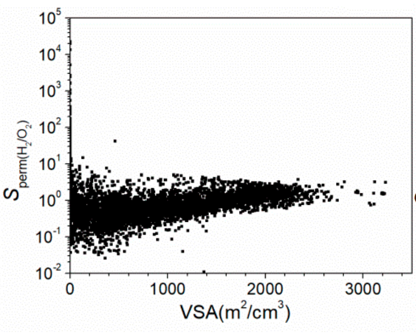

(c8) $S_{perm(H_2/O_2)}$ ~ VSA

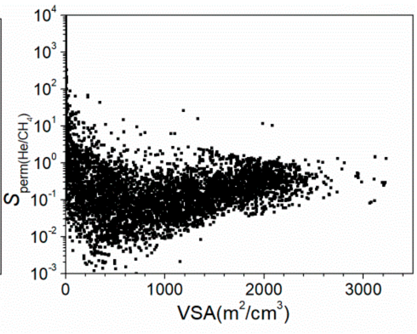

(c9) $S_{perm(He/CH_4)}$ ~ VSA

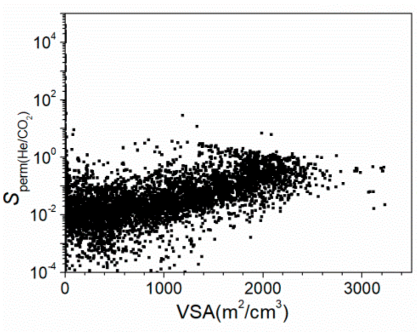

(c10) $S_{perm(He/CO_2)}$ ~ VSA

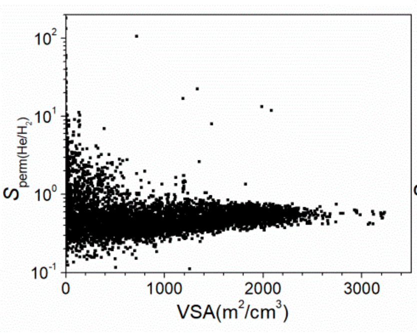

(c11) $S_{perm(He/H_2)}$ ~ VSA

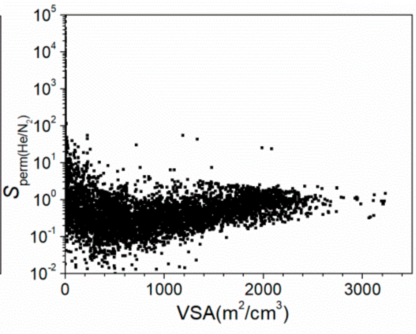

(c12) $S_{perm(He/N_2)}$ ~ VSA

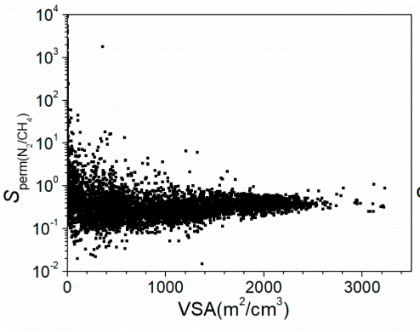

(c13) $S_{perm(N_2/CH_4)}$ ~ VSA

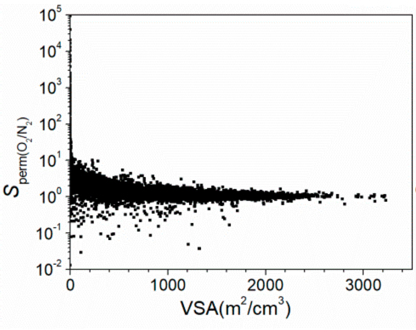

(c14) $S_{perm(O_2/N_2)}$ ~ VSA

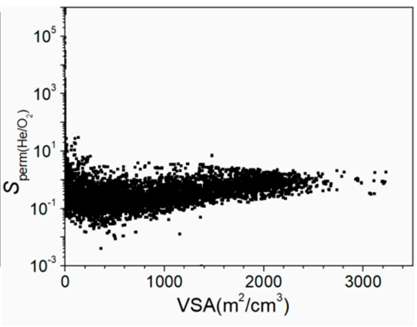

(c15) $S_{perm(He/O_2)}$ ~ VSA

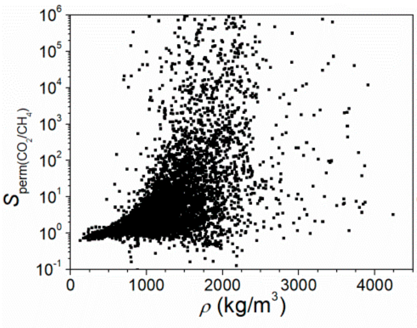

(d1) $S_{perm(CO_2/CH_4)}$ ~

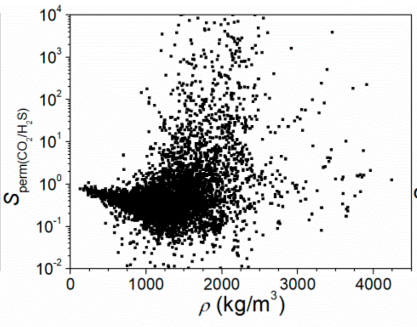

(d2) $S_{perm(CO_2/H_2S)}$ ~

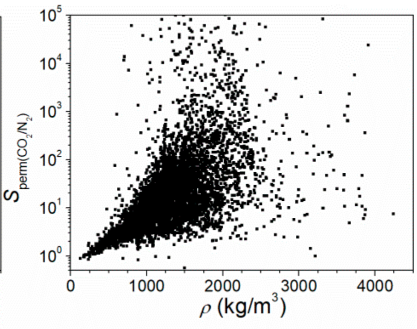

(d3) $S_{perm(CO_2/N_2)}$ ~

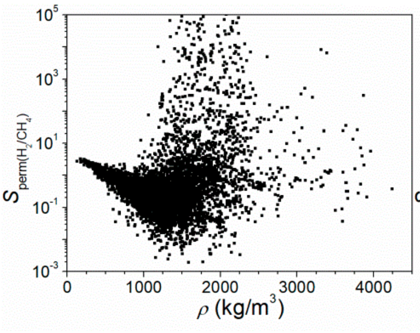

(**d4**) $S_{perm(H_2/CH_4)}$ ~

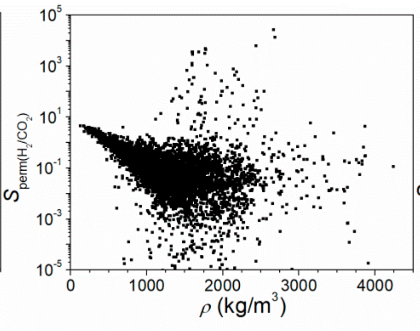

(**d5**) $S_{perm(H_2/CO_2)}$ ~

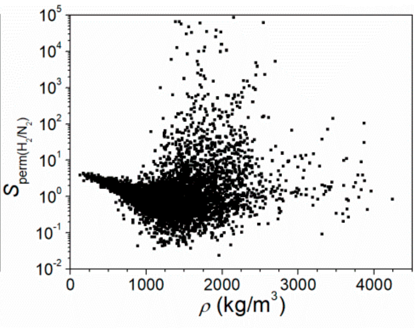

(**d6**) $S_{perm(H_2/N_2)}$ ~

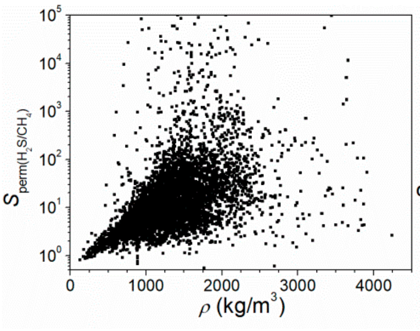

(**d7**) $S_{perm(H_2S/CH_4)}$ ~

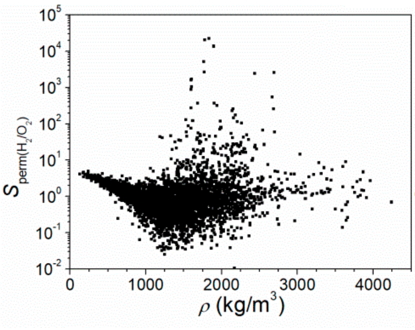

(**d8**) $S_{perm(H_2/O_2)}$ ~

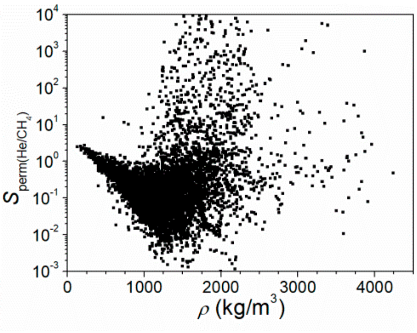

(**d9**) $S_{perm(He/CH_4)}$ ~

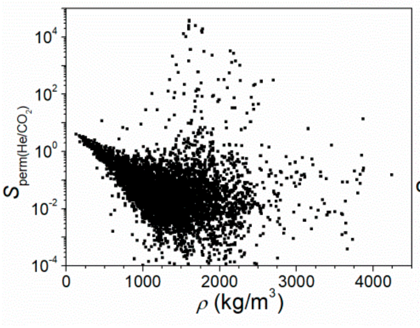

(**d10**) $S_{perm(He/CO_2)}$ ~

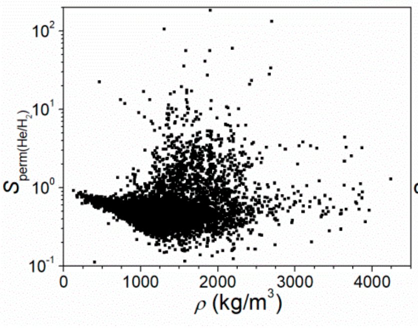

(**d11**) $S_{perm(He/H_2)}$ ~

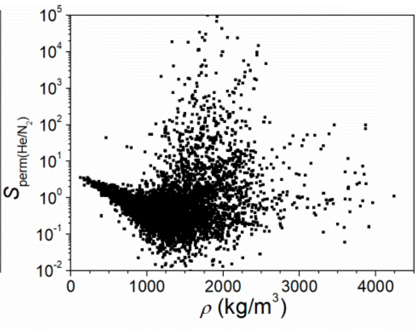

(**d12**) $S_{perm(He/N_2)}$ ~

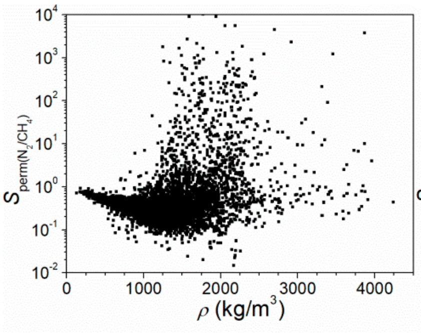

(**d13**) $S_{perm(N_2/CH_4)}$ ~

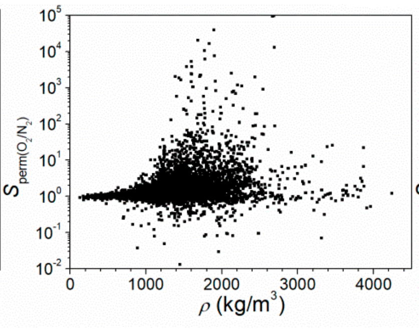

(**d14**) $S_{perm(O_2/N_2)}$ ~

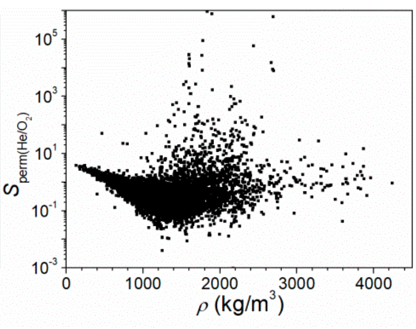

(**d15**) $S_{perm(He/O_2)}$ ~

Figure S5. Relationships between permselectivity S_{perm} and MOF descriptors: (**a**) S_{perm} and PLD, (**b**) S_{perm} and, (**c**) S_{perm} and VSA, (**d**) S_{perm} and, (1-15) represent 15 different gas mixtures (CO_2/CH_4,

CO_2/H_2S, CO_2/N_2, H_2/CH_4, H_2/CO_2, H_2/N_2, H_2S/CH_4, H_2/O_2, He/CH_4, He/CO_2, He/H_2, He/N_2, N_2/CH_4, O_2/N_2, and He/O_2).

Relationships between permeability P and permselectivity S_{perm}

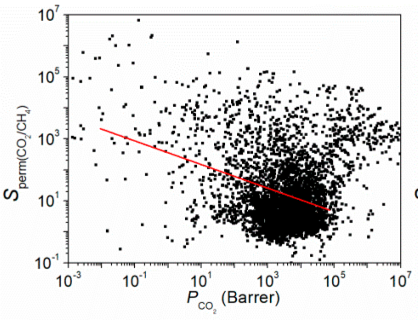

(**a**) P_{CO_2} ~ $S_{perm(CO_2/CH_4)}$

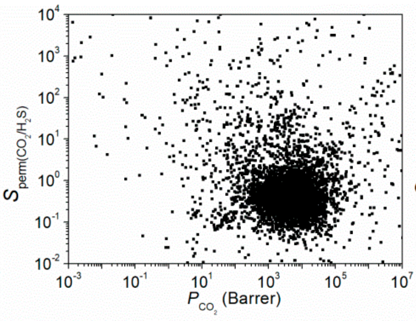

(**b**) P_{CO_2} ~ $S_{perm(CO_2/H_2S)}$

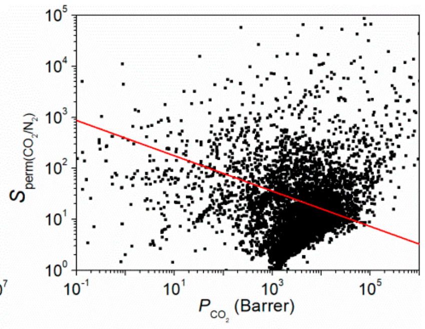

(**c**) P_{CO_2} ~ $S_{perm(CO_2/N_2)}$

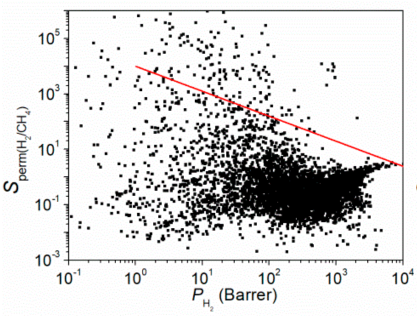

(**d**) P_{H_2} ~ $S_{perm(H_2/CH_4)}$

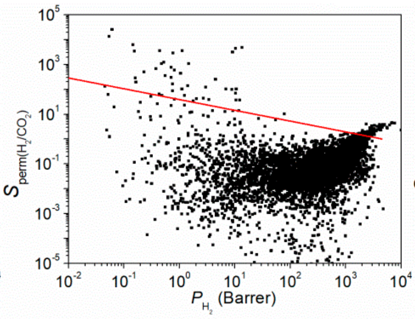

(**e**) P_{H_2} ~ $S_{perm(H_2/CO_2)}$

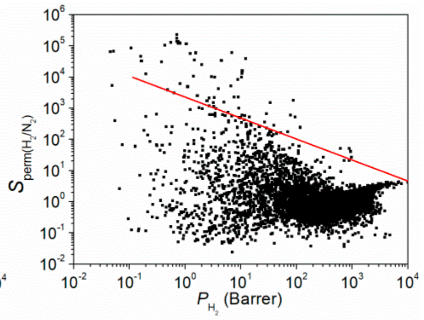

(**f**) P_{H_2} ~ $S_{perm(H_2/N_2)}$

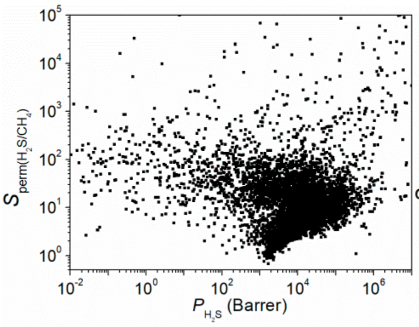

(**g**) P_{H_2S} ~ $S_{perm(H_2S/CH_4)}$

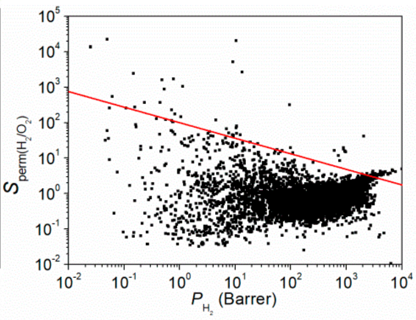

(**h**) P_{H_2} ~ $S_{perm(H_2/O_2)}$

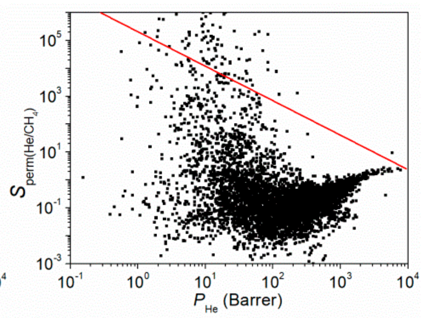

(**i**) P_{He} ~ $S_{perm(He/CH_4)}$

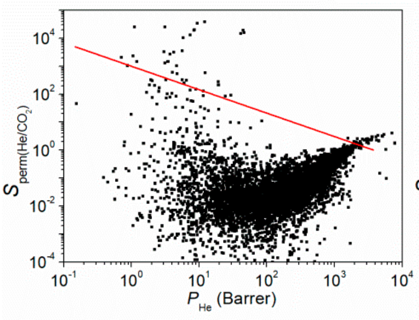

(**j**) P_{He} ~ $S_{perm(He/CO_2)}$

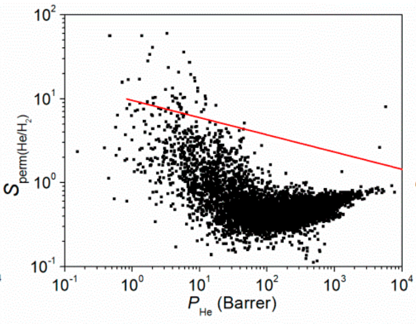

(**k**) P_{He} ~ $S_{perm(He/H_2)}$

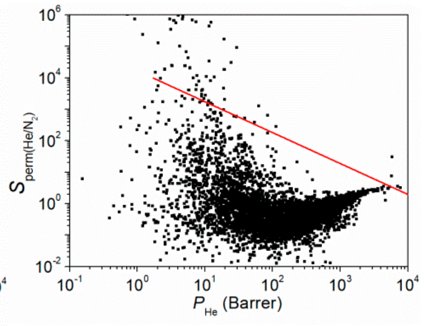

(**l**) P_{He} ~ $S_{perm(He/N_2)}$

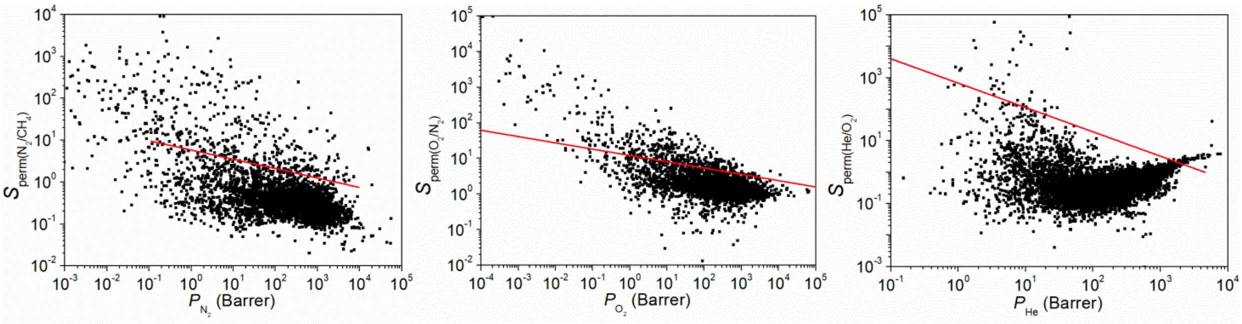

(**m**) P_{N_2} ~ $S_{perm(N_2/CH_4)}$ (**n**) P_{O_2} ~ $S_{perm(O_2/N_2)}$ (**o**) P_{He} ~ $S_{perm(He/O_2)}$

Figure S6. Relationships between permeability *P* and permselectivity S_{perm}, (**a–o**) represent 15 permanent gas mixtures (CO_2/CH_4, CO_2/H_2S, CO_2/N_2, H_2/CH_4, H_2/CO_2, H_2/N_2, H_2S/CH_4, H_2/O_2, He/CH_4, He/CO_2, He/H_2, He/N_2, N_2/CH_4, O_2/N_2, and He/O_2). The red line is the Robeson's upper bound for polymer membranes.

Table S3. Principal component covering the ratio of variation information for 44 performance metrics.

Principal component	PC1	PC2	PC3	PC4	PC5	PC6
Ratio of variation information	0.298	0.187	0.093	0.068	0.050	0.045
Principal component	PC7	PC8	PC9	PC10	PC1+PC2+...+PC10	
Ratio of variation information	0.042	0.032	0.030	0.024	0.870	

Table S4. RMSE and *R* versus four machine learning by synthesizing ten principal components

**Machine learning methods**	**DT**	**RF**	**SVM**	**BPNN**
RMSE	0.435	0.397	0.455	0.470
R	0.575	0.619	0.573	0.527

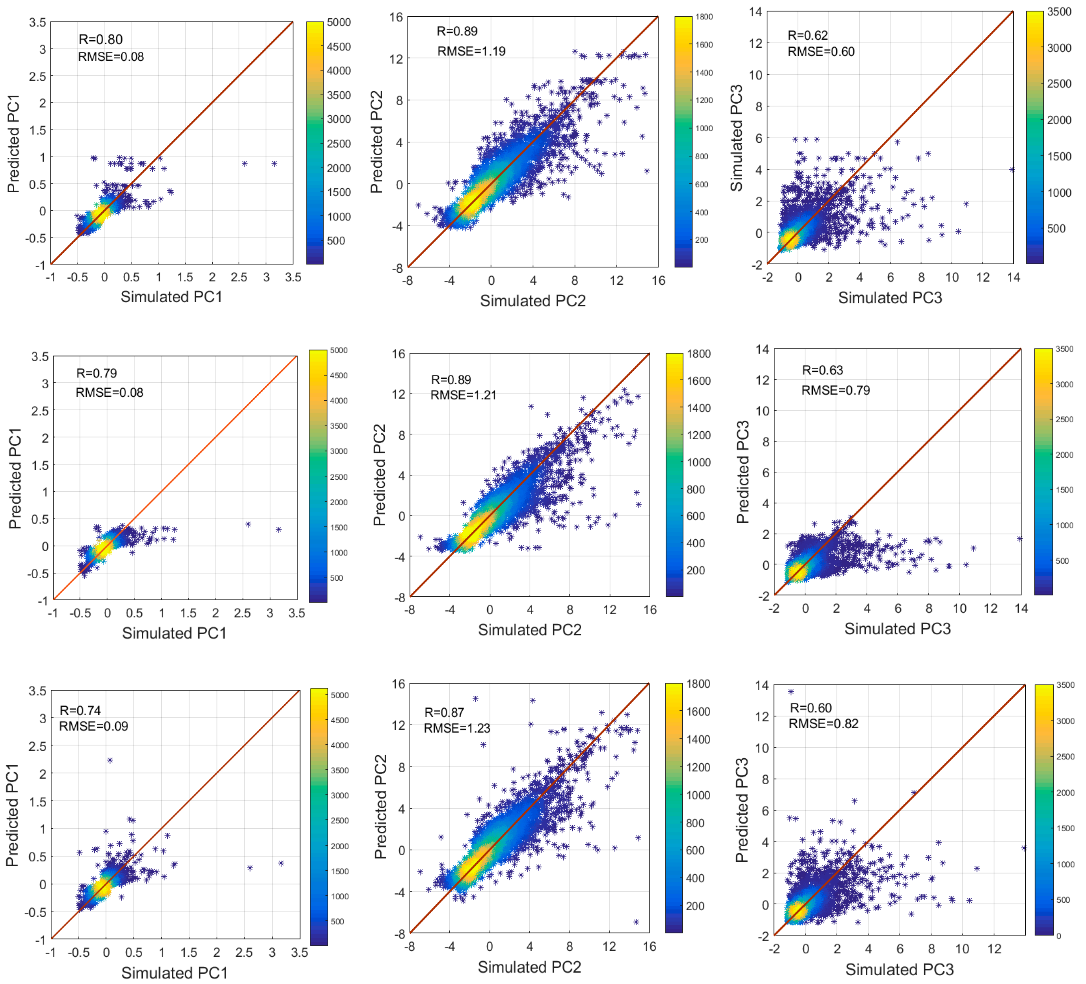

Figure S7. Predicted performance of the first three principal components (PC1, PC2, PC3) by three machine learning algorithm models of DT, SVM, and BPNN versus the simulated results of CoRE-MOFMs on the test set. The color of the point represents the amount of materials.

***k* times repeated *k*-fold cross-validation:**

k times repeated *k*-fold cross-validation is commonly used to evaluate the predicted performance on different predicted models. All of the data was randomly divided into *k*, where $k = 5$, in which one set was the test set, and the remaining four were training sets. The average of the root mean square error (RMSE) and the linear correlation coefficient (R) are regarded as an estimate index. This process is repeated five times.

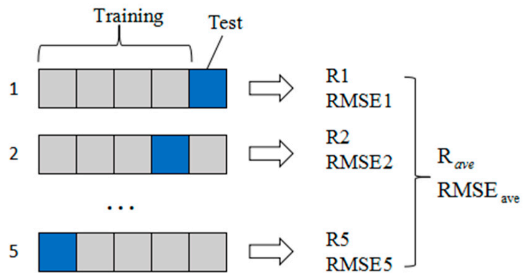

Principal Component Analysis

Principal component analysis (PCA) is commonly used for reducing a set of high-dimensional multivariate data to another set of low-dimensional space based on principal components without the loss of essential information. The principal components (PCs) are linear combinations of the original variables, and are thus orthogonal. In our study, the respective values of four descriptors (ϕ, Q^o_{st}, LCD, and VSA) are mean-centered and scaled to a unit variance, and then decomposed into a principal component score and eigenvector matrices via PCA procedure:

$$\boldsymbol{X} = \boldsymbol{T}\boldsymbol{P}^T + \boldsymbol{E} = \sum_{i=1}^{N} t_i\, p_i^T + \boldsymbol{E}$$

where $\boldsymbol{X}$ is the column-wise mean-centered and unit variance matrix, $\boldsymbol{T}$ is the principal component score matrix, $\boldsymbol{P}$ is the principal component eigenvector matrix, and $\boldsymbol{E}$ is the residual error matrix. If the original dataset has N different variables, PCA will give maximum N principal components. For each principal component i, t_i is the ith score column vector, and p_i is the ith eigenvector. The first few PCs are usually retained as they account for most of the data variance.

Decision tree (DT):

DT is a supervised learning method that could be used for both classification analyzing and regression predicting. The eigenvalues are selected from the most important to the next important in turn on the nodes of DT. Calculations are attempted for each eigenvalue on the DT algorithm model, followed by the attribution of the feature that would make the best classification as the parent node. Furthermore, the independent variable X_i is divided into two or more groups according to a certain splitting criterion. A tree is created through a plurality of splitting nodes. The binary branching is the most common DT. The independent variable X_i starts from the root node and is divided into two. Finally, DT is ended when it reaches the leaf nodes. The DT is created by an optimal splitting criterion, which makes the input Y_i as a test set the same as the input Y_i as a training set in leaf node, or makes the error of the input Y_i as a training set and the input Y_i as a test set within a specified error range.

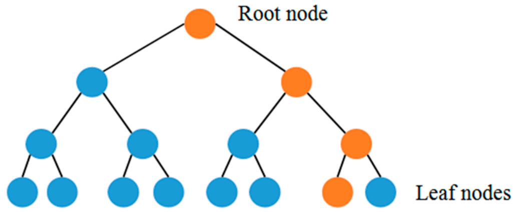

Random forest (RF):

RF is an improvement and optimization for DT, which is composed of multiple DTs. The n samples are randomly selected from independent variable X_i and k features are also randomly selected from all of the features in the RF algorithm, and the DT is established by the best segment feature attributes that are regarded as nodes. The 'm' DT are established through repeating steps 'm' times above. Therefore, the RF is composed of all DT. The response variable Y_i is predicted by R, which is, the average of inputting variable Y_i on the regression predicted for all the DT. The advantage of RF over the single DT is that the response variable Y_i could reduce the small change with the independent variable X_i changing slightly. In addition, RF could make up for the weakness of the generalization of DT and could grow from any type of DT.

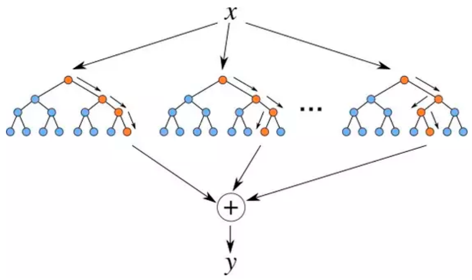

Support vector machine (SVM):

SVM is a supervised learning algorithm model that could be used for analyzing, pattern identifying, classifying, and regressing. The core of SVM is to map all the data points to a higher-dimensional space, and find an optimal surface in high-dimensional space. Therefore, each sample data is fitted into a linear model Y_i as much as possible in the training set. In addition, a tolerable constant ε (ε >0) is defined. When the absolute difference between the predicted Y_i' and the fitted Y_i is less than ε, that is regarded as no function loss. It is worth noting that the features of the SVM model is much smaller than the number of samples, and is sensitive enough to the missing data.

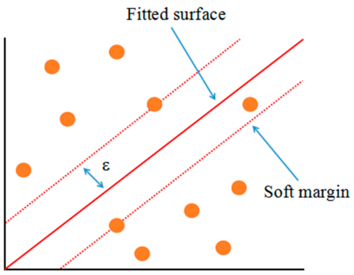

Back propagation neural network (BPNN).

BPNN is a kind of multilayer feedforward neural network whose main characteristics are signal forward and the error back propagation. In the signal forward, the input signal is handled step by step from the input layer through the hidden layer, until the output layer. Each layer of neurons state affects only the next layer of neurons state. If the output layer is not an expected consequence, it would be into the back propagation. According to the prediction error, it would adjust the network weights and thresholds automatically, so that the BP neural network closes to predict the output little by little. The essence of neural network learning is that the output error passes through reversely from the hidden layer to the input layer

step by step in some form; then, the output error spreads to all of the units in order to adjust the weights' dynamic by certain rules. The BP neural network topology structure as shown in the following:

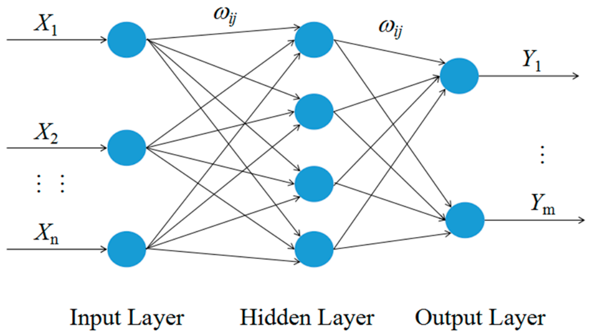

In the figure, X_1, X_2,…, X_n are the input values, Y_1, Y_2,... Y_m are the predictive values in the BPNN, and ω_j and ω_{jk} are the weights in the BPNN. As can be seen from Figure S5, the BPNN is a nonlinear function; the network input values and predicted values are the function of the independent variable and dependent variable, respectively. When inputting a node number as n, the output node number is m, and a functional mapping relationship is expressed from the dependent variables of n to the independent variables of m by the BPNN. When we predict different data by the ways of BPNN, first, the data are trained with associative memory and the ability to predict by network. When the network output error was reduced to an acceptable level or to the pre-set number of learning, it is terminated. Finally, the trained network is used to classify new data, fitting, and predicting.

Table S5. Benchmark of permeability and permselectivity for 15 gas mixtures.

i/j	CO_2/CH_4	CO_2/N_2	H_2/CO_2	H_2S/CH_4	H_2/CH_4	H_2/O_2	CO_2/H_2S	H_2/N_2	He/N_2	He/H_2	He/CH_4	N_2/CH_4	He/CO_2	O_2/N_2	He/O_2
P_i (Barrer)	2000	2000	2000	2000	800	2000	2000	2000	1000	20	100	500	30	800	2000
S_{perm}	10	10	3	5	3	3	100	3	3	3	10	10	3	3	3

Table S6. Best CoRE-MOFMs for different gas mixtures.

No.	*i/j*	CSD code	LCD (Å)	ϕ	VSA (m²/cm³)	PLD (Å)	ρ (g/m³)	PSD%	P_i (barrer)	P_j (barrer)	$S_{perm(i/j)}$
1	CO_2/CH_4	XUZDUS	4.25	0.16	15.50	2.99	1.82	3.31	1.26×10^9	1.75×10^3	7.16×10^5
		XEIXER	4.12	0.19	4.33	3.58	1.6	0	1.33×10^8	9.26×10^2	1.44×10^5
		ELUOIM06	2.89	0.04	0.00	2.41	1.8	100	7.20×10^6	6.44×10^3	1.12×10^3
		GETXAG	3.33	0.10	0.00	2.61	1.54	99.99	2.66×10^5	8.72×10^2	3.05×10^2
		CAHNEF	4.22	0.17	87.43	3.09	2.34	0.3	5.02×10^4	3.01×10^2	1.67×10^2
2	CO_2/N_2	ELUOIM06	2.89	0.04	0.00	2.41	1.8	100	7.20×10^6	7.72×10^2	9.32×10^3
		NHBZZN10	3.41	0.08	0.00	2.94	1.54	68.84	1.57×10^4	2.32×10^2	67.63
		FEJKEM	3.46	0.09	0.33	3.09	2.13	56.89	1.16×10^4	1.07×10^3	10.79
		SAPJIE	4.08	0.07	12.24	3.02	1.76	2.73	1.08×10^4	3.83×10^2	28.13
		YUBFUX	4.58	0.16	98.56	3.64	1.79	0	9.31×10^3	6.17×10^2	15.09
3	H_2/CO_2	TUMGOX	3.47	0.27	0	2.61	1.87	54.55	7.75×10^3	1.76×10^3	4.39
		HEDCEA	4.76	0.27	226.10	2.93	1.28	2.15	2.35×10^3	5.95×10^2	3.95
		LUSHUD	5.21	0.41	761.96	4.69	1.27	0	4.13×10^3	1.06×10^3	3.88
		EQERIC	5.65	0.56	1196.90	5.11	1.08	0	5.05×10^3	1.33×10^3	3.78
		FIHFAF	6.25	0.51	1116.42	4.37	1.21	0.04	6.23×10^3	1.68×10^3	3.71
4	H_2S/CH_4	SEYFAE	4.14	0.28	87.08	3.31	2.03	0.43	5.57×10^4	7.32×10^2	76.12
		GUXPUL	2.79	0.02	0.00	2.58	1.6	100	4.11×10^4	3.15×10^3	13.07
		PARFOF	2.77	0.05	0.00	2.46	1.54	100	1.92×10^4	1.79×10^3	10.70

		OBENUF	3.75	0.16	0.33	3.19	2.05	0.12	8.56×10^{3}	2.68×10^{2}	31.91
		FIHXUR	3.33	0.05	0.00	2.95	1.46	100	1.72×10^{3}	82.34	20.94
5	H_2/CH_4	TESGUU	4.82	0.26	338.16	3.58	1.92	0	8.90×10^{2}	0.07	1.20×10^{4}
		ZIJVOF	5.29	0.43	622.86	3.32	1.23	0.10	9.35×10^{2}	0.10	9.37×10^{3}
		VUQKOI	6.97	0.35	874.66	5.61	1.36	0.07	8.95×10^{2}	0.13	6.67×10^{3}
		POBYAH	5.61	0.27	768.04	3.91	3.11	0.10	2.08×10^{3}	15.77	1.31×10^{2}
		PIYFIO	6.28	0.40	1129.69	2.79	1.81	0	8.52×10^{2}	2.03×10^{2}	4.20
6	H_2/O_2	TOWPAY	3.4731	0.27	0	2.61	1.87	54.55	7.75×10^{3}	1.89×10^{3}	4.10
		FIHFAF	6.25	0.51	1116.42	4.37	1.21	0.04	6.23×10^{3}	1.67×10^{3}	3.74
		POWBIO	4.34	0.19	142.64	2.60	3.66	1.54	4.56×10^{3}	1.18×10^{3}	3.87
		SABWAU	2.84	0.11	0	2.43	1.46	100.00	4.37×10^{3}	1.31×10^{3}	3.34
		LAGMUD	4.81	0.54	1363.40	3.83	1.13	0.01	4.35×10^{3}	1.20×10^{3}	3.63
7	CO_2/H_2S	FAPYEA	2.53	0.00	0.00	2.46	1.58	100	1.93×10^{9}	5.11×10^{5}	3.78×10^{3}
		XUZDUS	4.25	0.16	15.50	2.99	1.82	3.31	1.26×10^{9}	6.80×10^{6}	1.85×10^{2}
		KIJYIM	3.01	0.17	0.00	2.52	2.79	99.99	2.92×10^{4}	7.14	4.08×10^{3}
		JOCPIB	3.52	0.06	0.00	2.59	1.77	99.99	2.49×10^{4}	3.22	7.74×10^{3}
		WOCVUG01	4.33	0.17	57.26	3.42	1.5	1.3	9.10×10^{3}	7.05	1.29×10^{3}
8	H_2/N_2	TUMGOX	3.47	0.27	0	2.61	1.87	54.55	7.75×10^{3}	2.01×10^{3}	3.85
		FIHFAF	6.25	0.51	1116.42	4.37	1.21	0.04	6.23×10^{3}	1.73×10^{3}	3.60
		FAZFET	6.57	0.43	1272.81	4.46	1.65	0.21	4.46×10^{3}	1.26×10^{3}	3.55
		DIMQOH	4.69	0.42	626.81	3.15	1.40	2.97	3.80×10^{3}	1.12×10^{3}	3.39
		ICANAD	7.32	0.32	428.55	2.77	2.01	0.00	3.58×10^{3}	1.17×10^{3}	3.05
9	He/N_2	TUMGOX	3.47	0.27	0	2.61	1.87	54.55	7.11×10^{3}	2.01×10^{3}	3.53
		EQERIC	5.65	0.56	1196.90	5.11	1.08	0.00	4.37×10^{3}	1.25×10^{3}	3.50
		COWXOC	5.83	0.30	517.90	2.91	1.28	0.57	7.79×10^{3}	2.43×10^{3}	3.21
		SAHYOQ03	15.06	0.82	2101.62	7.94	0.59	0	2.70×10^{3}	8.72×10^{2}	3.10
		JENKIX	9.92	0.50	1084.71	7.49	1.01	0	3.61×10^{3}	1.19×10^{3}	3.02
10	He/H_2	DUOCAU01	4.78	0.28	335.23	2.43	2.20	0.04	45.34	9.16	4.95
		EMITUQ	7.80	0.60	1446.69	5.62	0.97	0.04	44.79	10.43	4.30
		UFUQIV	7.38	0.70	1858.64	5.82	0.68	0	37.19	9.29	4.00
		BUYNAL	4.62	0.47	617.88	3.97	0.92	0	20.34	5.12	3.97
		VULKOD	5.02	0.33	489.24	3.96	1.40	0.95	25.60	6.52	3.93
11	He/CH_4	GAXGET	4.84	0.24	146.96	3.01	1.34	1.26	1.10×10^{2}	0.01	7.51×10^{3}
		YEKWOC	8.21	0.50	1099.57	3.05	1.28	0	1.21×10^{2}	0.11	1.15×10^{3}
		COXFOL	5.01	0.33	403.58	3.19	1.43	1.70	3.24×10^{2}	7.48	43.32
		XOFTIW	3.88	0.08	5.89	3.29	2.44	10.41	1.01×10^{2}	5.63	17.98
		WIFGOJ	9.99	0.30	623.13	2.45	1.99	0.60	1.74×10^{2}	11.94	14.56
12	N_2/CH_4	YEKWOC	8.21	0.50	1099.57	3.05	1.28	0	6.82×10^{3}	0.11	6.44×10^{4}
		BAHGUN04	4.27	0.26	108.86	3.29	1.49	0	6.24×10^{5}	11.10	5.62×10^{4}
		POBYAH	5.61	0.27	768.04	3.91	3.11	0.10	3.06×10^{5}	15.77	1.94×10^{4}
		GOSDEZ	8.09	0.59	1562.91	5.76	0.99	0	6.19×10^{2}	10.57	58.56
		JEMNAR	5.06	0.36	349.97	3.17	1.50	0.18	7.09×10^{2}	43.03	16.48
13	He/CO_2	EMITUQ	7.80	0.60	1446.69	5.62	0.97	0.04	44.79	2.37×10^{-3}	1.89×10^{4}
		DUQCAU01	4.78	0.28	335.23	2.43	2.20	0.04	45.34	2.89×10^{-3}	1.57×10^{4}
		PUPNAQ	3.59	0.15	0	2.70	1.44	32.37	38.34	4.11	9.33
		XEQRAO	5.51	0.28	601.41	4.61	1.37	0	57.28	9.36	6.12
		TUMGOX	3.47	0.27	0	2.61	1.87	54.55	7.11×10^{3}	1.76×10^{3}	4.03
14	O_2/N_2	XOTXAG	4.59	0.42	686.91	3.72	1.46	0.09	2.16×10^{3}	5.82×10^{2}	3.72
		NIKZAJ02	4.67	0.25	353.58	3.69	1.77	0	1.05×10^{3}	2.82×10^{2}	3.71
		GETXAG	3.33	0.10	0	2.61	1.54	99.99	1.11×10^{4}	3.17×10^{3}	3.48
		GOLQII	3.73	0.14	9.74	3.37	2.17	0	8.32×10^{2}	2.48×10^{2}	3.35
		HIFZAY	4.52	0.17	107.91	3.46	1.49	1.54	1.14×10^{3}	3.47×10^{2}	3.27
15	He/O_2	COWXOC	5.83	0.30	517.90	2.91	1.28	0.57	7.79×10^{3}	2.07×10^{3}	3.76
		TUMGOX	3.47	0.27	0	2.61	1.87	54.55	7.11×10^{3}	1.89×10^{3}	3.76
		FIHFAF	6.25	0.51	1116.42	4.37	1.21	0.04	5.12×10^{3}	1.67×10^{3}	3.07
		EOERIC	5.65	0.56	1196.90	5.11	1.08	0	4.37×10^{3}	1.23×10^{3}	3.55
		FORWAL	6.10	0.40	687.66	3.43	1.14	0	2.29×10^{3}	7.19×10^{2}	3.18

References

1. Rappe, A.K.; Casewit, C.J.; Colwell, K.S.; Goddard, W.A.; Skiff, W.M. UFF: A Full Periodic Table Force Field for Molecular Mechanics and Molecular Dynamics Simulations. *J. Am. Chem. Soc*. **1992**, *114*, 10024–10035.

2. Martin, M.G.; Siepmann, J.I. Transferable Potentials for Phase Equilibria. 1. United-Atom Description of n-Alkanes. *J. Phys. Chem. B.* 1998,102:2569–2577.

3. Shah, M. S.; Tsapatsis, M.; Siepmann, J. I. Development of the Transferable Potentials for Phase Equilibria Model for Hydrogen Sulfide. *J. Phys. Chem. B.* 2015,119:7041−7052.

4. Küsgens, P.; Rose, M.; Senkovska, I.; Fröde, H.; Henschel, A.; Siegle, S.; Kaskel, S. Characterization of metal-organic frameworks by water adsorption. *Microporous Mesoporous Mater.* **2009**, *120*, 325–330.
5. Hayter, A. J. *Probability and Statistics for Engineers and Scientists*. 4th Ed.; Cengage Learning: Ohio, 2012.
